# Extra-hematopoietic immunomodulatory role of the guanine-exchange factor DOCK2

**DOI:** 10.1038/s42003-022-04078-1

**Published:** 2022-11-15

**Authors:** Cornelia Scharler, Rodolphe Poupardin, Patricia Ebner-Peking, Martin Wolf, Christina Schreck, Gabriele Brachtl, Andre Cronemberger Andrade, Linda Krisch, Laurence Daheron, Katharina Schallmoser, Karsten Jürchott, Judit Küchler, Harald Stachelscheid, Hans-Dieter Volk, Robert A. J. Oostendorp, Dirk Strunk

**Affiliations:** 1 Cell Therapy Institute, Spinal Cord Injury and Tissue Regeneration Center (SCI-TReCS), Paracelsus Medical University (PMU), Salzburg, Austria; 2 Technical University of Munich, School of Medicine, Internal Medicine III, Munich, Germany; 3 Department of Transfusion Medicine and SCI-TReCS, PMU, Salzburg, Austria; 4 HSCI iPS Core Facility, Harvard University, Cambridge, USA; 5 BCRT & Institute of Medical Immunology, Charité - Universitätsmedizin Berlin, Berlin, Germany

**Keywords:** Medical research, Immunology

## Abstract

Stromal cells interact with immune cells during initiation and resolution of immune responses, though the precise underlying mechanisms remain to be resolved. Lessons learned from stromal cell-based therapies indicate that environmental signals instruct their immunomodulatory action contributing to immune response control. Here, to the best of our knowledge, we show a novel function for the guanine-exchange factor DOCK2 in regulating immunosuppressive function in three human stromal cell models and by siRNA-mediated DOCK2 knockdown. To identify immune function-related stromal cell molecular signatures, we first reprogrammed mesenchymal stem/progenitor cells (MSPCs) into induced pluripotent stem cells (iPSCs) before differentiating these iPSCs in a back-loop into MSPCs. The iPSCs and immature iPS-MSPCs lacked immunosuppressive potential. Successive maturation facilitated immunomodulation, while maintaining clonogenicity, comparable to their parental MSPCs. Sequential transcriptomics and methylomics displayed time-dependent immune-related gene expression trajectories, including DOCK2, eventually resembling parental MSPCs. Severe combined immunodeficiency (SCID) patient-derived fibroblasts harboring bi-allelic DOCK2 mutations showed significantly reduced immunomodulatory capacity compared to non-mutated fibroblasts. Conditional DOCK2 siRNA knockdown in iPS-MSPCs and fibroblasts also immediately reduced immunomodulatory capacity. Conclusively, CRISPR/Cas9-mediated DOCK2 knockout in iPS-MSPCs also resulted in significantly reduced immunomodulation, reduced CDC42 Rho family GTPase activation and blunted filopodia formation. These data identify G protein signaling as key element devising stromal cell immunomodulation.

## Abstract (web summary)

The guanine exchange factor DOCK2 regulates the development of immunomodulatory function in human stromal cells (mesenchymal stem/progenitor cells (MSPC) and fibroblasts), as demonstrated in three models.

## Introduction

Effective control limiting or ending immune responses is of utmost importance. Specialized effector lymphocytes educated in primary and secondary lymphoid organs execute immunity. Dendritic cells adjust immunity and interact with regulatory T cells in immune control^1^. The multifaceted contribution of stromal cells to immune modulation is widely neglected^2^. Stromal cells are considered as connective tissue elements comprising resident fibroblasts and various mural cell types around blood vessels. Pericytes surround smaller capillaries whereas smooth muscle cells stabilize larger vessels^3^. Multiple types of MSPCs maintain the organ-specific stromal cell populations. Their well-recognized role in tissue homeostasis and regeneration motivated a multiplicity of cell-based therapy applications in regenerative medicine^4^. When tested in vitro, however, virtually all stromal cell types additionally inhibit immune functions to variable extent^5–7^. The appealing concept of ‘mesenchymal stem cells’ initiating a ‘mesengenic process’ was proposed based on in vitro data on multi-lineage osteo-, chondro- and adipogenic differentiation of bone marrow stromal cells^8,9^. A plethora of distinct MSPC types, mostly lacking ‘stemness’ features, was meanwhile identified in bone marrow^10^, skin^11^, and other tissue^12^. Their peculiar immune function remains largely enigmatic^12,13^. The diverse immunomodulatory properties of MSPCs led to the initiation of multiple clinical trials exploring their mainly trophic secretory function to treat multiple inflammatory conditions, but their precise mode of action remains to be resolved^5,14,15^. High expectations were so far not satisfied in clinical trials determining MSPCs’ immunomodulatory and trophic effects^15,16^. Resurgent interest in stromal immunomodulatory function was attracted by observations indicating a complex interplay between regulatory T cells and stromal cells as potent immune response inhibitors^17^. Excitingly, thymus fibroblasts were recently recognized to regulate central tolerance by providing self-antigens during negative selection of autoreactive T cells in vivo^18^.

Here we employed ‘closed circles’ of autologous oligoclonal MSPC-to-iPS-back-to-MSPC differentiation, derived from bone marrow (BM) and umbilical cord blood (UCB), to mimic stromal ontogeny approaching mature immunomodulatory phenotype and function. Stringent omics, without the common ‘noise’ due to donor and organ variability, identified an unexpectedly high proportion of immune-related and small G protein-coupled receptor (GPCR) signaling genes, including the DOCK2-RAC2 GTPase axis, accompanying stromal immune maturation. We confirmed these findings in two independent proof-of-concept models. We further demonstrate that fibroblasts from DOCK2-mutated SCID patients show defective immunosuppression associated with reduced CDC42-dependent filopodia formation, indicating that DOCK2 GTPase activation is necessary for immunosuppressive MSPC function. CRISPR-Cas9 knockout in control iPSCs, in advance of MSPC maturation, confirmed the role of DOCK2 during stromal immune function. DOCK2 knockdown by siRNA also immediately reduced the immunomodulatory potential of skin fibroblasts and iPS-MSPCs compared to mock-treated controls. This adds a novel facet to the sequence of events through which stromal cells suppress T cell proliferation and opens up countless opportunities for validating GPCRs as therapeutic targets in stromal immunomodulation.

## Results

**Fig. 1:**
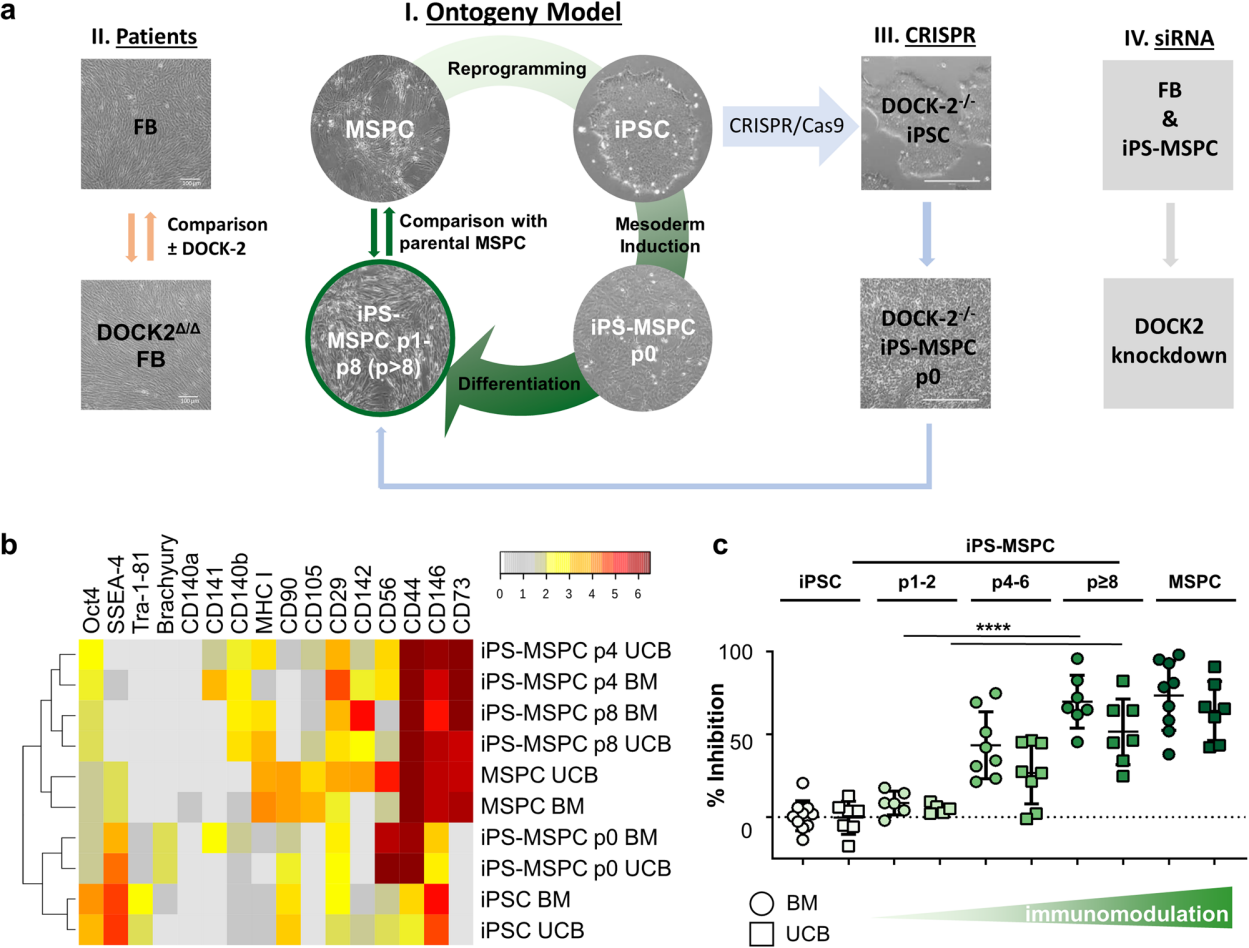
Human iPS-MSPCs undergo continuous maturation towards reacquisition of immune modulatory potential. **a** Experimental setup: **I**. MSPCs derived from bone marrow (BM) and umbilical cord blood (UCB) were reprogrammed into iPSCs before mesoderm induction and maturation into mesodermal iPS-MSPCs to enable sequential gene expression and functional profiling. **II**. DOCK-2 mutated patient fibroblast (FB) lines were compared to healthy controls for phenotype and immunomodulation. **III**. Healthy iPSCs were used for CRISPR/Cas9-mediated bi-allelic DOCK-2 knockout, subsequent DOCK-2^-/-^ iPS-MSPC propagation and function comparison. **IV**. Healthy control FB and iPS-MSPCs were subjected to si-RNA-mediated DOCK2 knockdown to determine the immediate effect of reduced DOCK2 GEF presence in stromal cells during immunomodulation. **b** Cluster analysis of single cell-based flow cytometry marker profiling during differentiation of MSPC-derived iPSCs back into iPS-MSPCs. Mean ΔMFI values (specific antibody staining minus isotype control) from passage 0 (p0) to p8, compared to parental MSPCs. **c** Immunomodulatory potential of iPS-MSPCs inhibiting T-cell mitogenesis at 1:3 ratio iPSCs or MSPCs:immune cells increased with differentiation from early to late passages compared to parental primary MSPCs and iPSCs. (BM: iPSC *n* = 10, iPS-MSPC p1 – 2 *n* = 6, iPS-MSPC p4 – 6 *n* = 8, iPS-MSPC *p* ≥ 8 = 7, MSPC *n* = 9; UCB: iPSC *n* = 7, iPS-MSPC p1 – 2 *n* = 5, iPS-MSPC p4 – 6 *n* = 8, iPS-MSPC *p* ≥ 8 *n* = 7, MSPC *n* = 7; PBMC:MSPC ratio 1:3; one-way ANOVA including Tukey´s multiple comparison test: ****p < 0.0001 for BM and UCB, BM: df = 70, *F* = 24.16; UCB: df = 58, *F* = 25.48). Percent inhibition after normalizing mean T cell proliferation values of individual assays shown. Symbol and color-code as indicated. Error bars represent standard deviation.

We devised mesodermal stromal maturation from iPSCs using an ontogeny-inspired model system to understand stromal immune functions. Parental MSPCs served as source for reprogramming and as reference for determining phenotypic and functional maturation of iPS-MSPCs. We selected primary human MSPCs from BM and UCB representing two different tissues of origin as well as adult and juvenile sources for reprogramming. Both oligoclonal parental BM- and UCB-MSPC strains were confirmed to inhibit T cell proliferation dose-dependently as surrogate of their immunomodulatory function^7^. We next reprogrammed these primary MSPCs into iPSCs using a non-integrative Sendai virus protocol^19^ with transient expression of reprogramming factors Oct4, Sox2, KLF4 and c-Myc^20^. The MSPC-derived iPSCs were subjected to consecutive differentiation and maturation, first towards mesoderm (passage zero, p0) and then along mesodermal stromal/fibroblast lineage, to study immunophenotype and functional maturation compared to parental MSPCs (Fig. 1a I). Progressive pluripotency marker decline was paralleled by fibroblastic marker acquisition during iPSC-into-iPS-MSPC differentiation (Fig. 1b, Supplementary Fig. 1). The stromal cell-inherent immunosuppressive capacity was analyzed in some but not all previous studies on iPSC-derived MSPCs (summarized in Supplementary Table 1). We observed a gradual significant increase of MSPC-mediated inhibition of T cell proliferation with maturation from iPSC-to-iPS-MSPCs eventually matching parental MSPCs’ immunosuppression capacity (Fig. 1c).

Human BM-MSPC- and UCB-MSPC-derived iPSCs showed a normal karyotype after Sendai virus elimination and spontaneously differentiated into endodermal, ectodermal and mesodermal tissue forming teratomas in immunodeficient mice (Supplementary Fig. 2a–c). We first induced mesoderm from four BM-derived clones (PMUi001-A-D) and three UCB-derived clones (PMUi002-A-C; https://hpscreg.eu/) as confirmed by CD56 and brachyury upregulation (Supplementary Fig. 3)^21^. Oct4, Tra-1-81 and SSEA-4 pluripotency marker expression decreased during differentiation. Brachyury and CD56 were most abundant in iPS-MSPCp0 immediately after mesoderm induction (Fig. 1b, Supplementary Fig. 1 + Supplementary Fig. 3). Significant canonical stromal marker upregulation including CD73 (5’-nucleotidase) and CD105 (endoglin), and reduction of Tra-1-81 during differentiation (Supplementary Fig. 1), was paralleled by consecutive acquisition of typical fibroblastic morphology comparable to parental MSPCs (Supplementary Fig. 4). Reproducible gain of adhesion molecule (CD146)^22^, platelet-derived growth factor receptor-beta (CD140b)^23^; and tissue factor (CD142)^22^ expression was evident during iPS-MSPC maturation, separating MSPCs from iPSCs and early mesoderm (p0; Fig. 1b). Integrin-β1 (CD29), phagocytic-glycoprotein-1 (CD44) and major histocompatibility complex class I (MHC I) indicated progressive iPS-MSPC maturation (Fig. 1b and Supplementary Fig. 1). Clonogenicity was maintained and trilineage differentiation capacity was re-acquired, with restricted chondrogenic differentiation compared to parental BM-MSPCs^24^ (Supplementary Fig. 5). We confirmed both parental MSPC strains inhibiting mitogen-induced CD3 T cell proliferation dose-dependently. Reprogramming into iPSCs was paralleled by a complete loss of immunomodulation. Early passage iPS-MSPCs also lacked immunomodulatory capacity. Starting after four passages, iPS-MSPCs significantly inhibited T cell proliferation. The maximum level of immunomodulation was reached beyond passage eight, representing inhibition strength to autologous parental MSPCs (Fig. 1c and Supplementary Fig. 6).

**Fig. 2:**
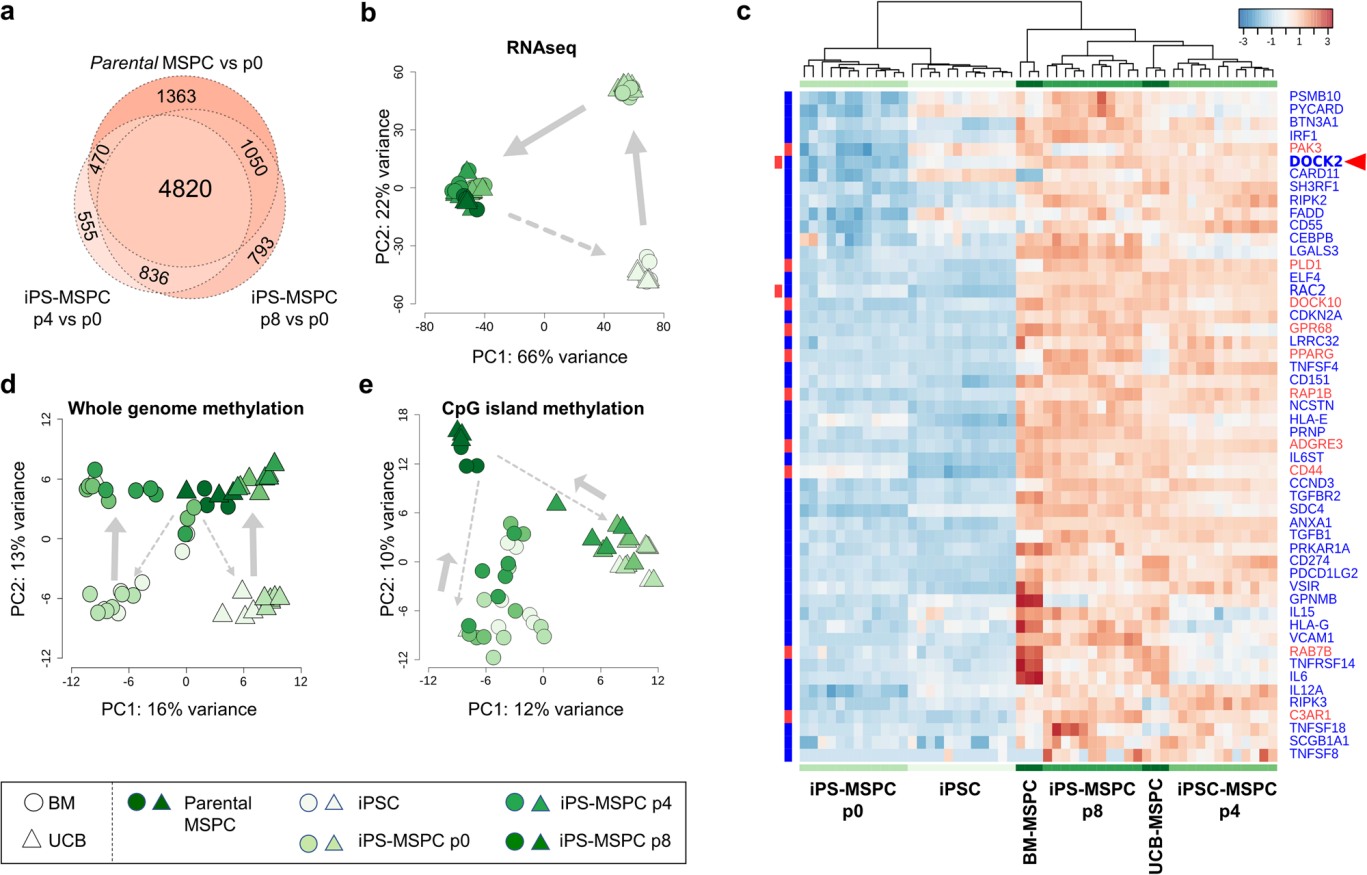
Sequential methylome and gene expression analysis during MSPC-to-iPS-to-MSPC ontogeny. **a** Venn diagram summarizing the number of significantly >2-fold differentially regulated genes in parental MSPCs vs. iPS-MSPCs p0 and iPS-MSPCs p4 vs. p0 and p8 vs. p0, respectively (adjusted *p*-value < 0.05). **b** Principal component (PC) transcriptome analysis. **c** Hierarchical clustering heat map of genes linked with T-cell proliferation () or G-protein signaling () found significantly upregulated in iPS-MSPCp8 vs. p0 and also represented in parental MSPCs. **d** Whole methylome levels and **e** CpG island methylation sub-analysis showing relationship between different samples. Gray arrows indicating differentiation trajectories. *p*-values were adjusted using Benjamini-Hochberg correction. (RNA-seq: iPSCs, *n* = 12; p0, *n* = 12; p4, *n* = 12; p8, *n* = 11; parental MSPCs, *n* = 6; MethylCap-seq: iPSCs, *n* = 11; p0, *n* = 12; p4, *n* = 9; p8, *n* = 10; parental MSPCs, *n* = 6).

This ‘closed circle’ of MSPC-to-iPS-back-to-MSPC ontogeny enabled stringent autologous gene expression profiling of multiple clones by RNA sequencing at key differentiation steps (Fig. 1a I), compared to parental MSPCs. Comparing immune-competent (parental MSPCs, iPS-MSPCp4, iPS-MSPCp8) with immune-refractory cells (iPS-MSPCp0), we identified 4,820 genes, representing 25% of the 19,287 detectable gene IDs, commonly differentially regulated across MSPC ontogeny (Fig. 2a). Whole transcriptome principal component (PC) analysis identified a tremendously stable developmental process covering 66% and 22% gene expression variance in two dominant gene clusters, PC1 and PC2, respectively (Fig. 2b), demonstrating reacquisition of parental MSPC gene expression profiles (Fig. 2c). Likewise, distinct trajectories of whole-genome methylation changes (Fig. 2d) and CpG island methylation (Fig. 2e) occurred during reprogramming and subsequent immune-competent stromal cell re-differentiation recapitulating MSPC ontogeny, despite effecting a more restricted gene set. An incomplete methylome identity retrieval at p0, compared to autologous parental MSPCs, corresponded to the lack of complete surface marker identity, e.g., diminished CD90 and SSEA-4. Further phenotypic maturation was, however, achieved beyond p8^25^ (Supplementary Fig. 9c), not covered by omics in this study.

When comparing differentially methylated regions between p0 and p8, CpG islands and transcription start sites showed strongest gains and losses (Supplementary Fig. 7a). Telomere regions were differential methylation hotspots (Supplementary Fig. 7b) consistent with epigenetic determination of their identity and function^26^. Pronounced CpG island methylation changes were observed during iPSC-derived mesodermal stromal ontogeny, despite partial re-establishment of parental epigenomic signatures^27^. Panther analysis showed enrichment of genes involved in T cell proliferation and GPCR signaling (Fig. 2c), but none of the 53 selected predominantly regulated genes were significantly differentially methylated (Supplementary Fig. 7c, d). This suggests regulatory mechanisms other than methylation being involved, although we cannot exclude small methylation differences, not detected by MethylCap-seq of samples from different donors, to be operative. Remarkably, the top 196 differentially methylated genes (Fig. S7b), showed a gene ontology (GO) term enrichment of ‘regulation of GTPase activity’ and ‘regulation of small GTPase mediated signal transduction’ (Supplementary Fig. 7c). Most of these GTPase-associated genes were hypomethylated (Supplementary Fig. 7b). We found 51 additional immune response-related genes differentially methylated and expressed in BM-derived iPS-MSPCp8 and 160 genes in UCB-derived iPS-MSPCp8 vs p0, respectively. Interestingly, 44 genes found in both comparisons suggest that immune function acquisition from both sources partially shared similar methylation patterns, including EphB2 among most upregulated and hypomethylated genes. We further found the separation of BM- and UCB-derived iPSCs and iPS-MSPCp0 at methylation level, in contrast to RNA-sequencing where gene expression of both cell sources clustered together throughout differentiation. The current study did not discriminate source-related vs. donor-derived changes (Fig. 2d, e, Supplementary Fig. 7c).

The top 500 genes regulated over time reflected the developmental path of iPS-MSPCs re-approaching their adult parental predecessor’s gene expression profile (Supplementary Fig. 8a). Among the 200 most significant upregulated ‘immune system process’ genes we focused on those 25% related to T cell proliferation (Supplementary Fig. 8b). We found two striking gene clusters regulating T cell proliferation and immune-related GPCR signaling (Fig. 2c, Supplementary Table 2). *DOCK-2* attracted our attention because mutations in this guanine-exchange factor (GEF), commonly involved in Rho kinase regulation, resulted in combined immunodeficiency^28,29^. This may point to a missing link between immune cell dysfunction and the still enigmatic immune response modulation by stromal niche cells^5^. Additional regulation of Ras-related botulinum-toxin substrate-2 (RAC2) and further G-protein signaling molecules during iPS-MSPC ontogeny connected DOCK-2 with stromal immunomodulation (Fig. 2c, Supplementary Table 2).

**Fig. 3:**
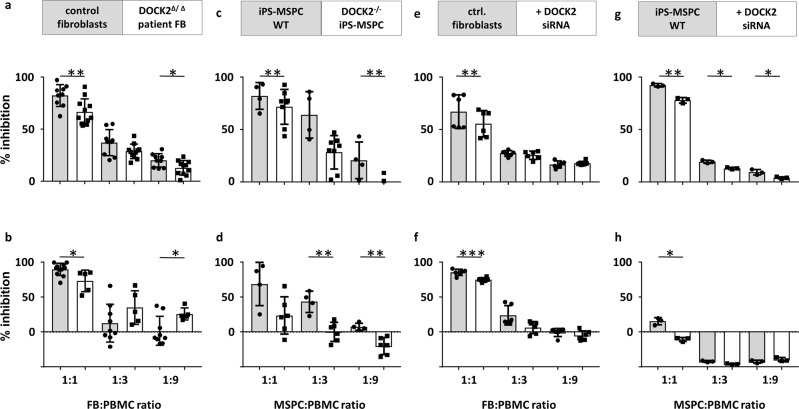
DOCK2-deficient (Δ/Δ) patient fibroblasts, CRISPR/Cas9-created DOCK2-/- iPS-MSPCs and siRNA-mediated DOCK2 knockdown impaired stromal immunomodulation. (**a**, **c**, **e**, **g**) T cell mitogenesis and (**b**, **d**, **f**, **h**) allogeneic mixed leukocyte reaction (MLR). **a** Pooled data from two *DOCK2*^Δ/Δ^ patient’s fibroblasts and four different adult donor’s fibroblasts (control fibroblasts 1:1 *n* = 9, 1:3 *n* = 9, 1:9 *n* = 9, DOCK2-deficient fibroblasts 1:1 *n* = 11, 1:3 *n* = 11, 1:9 *n* = 11, for 1:1 **p < 0.01, unpaired two-tailed *t* test *p*-value 0.0077, df = 54, 1:9 *p* = 0.029, df = 18). **b** MLR at day 7. Pooled data of the two DOCK2 patients, 5 different adult fibroblast and 2 neonatal fibroblast donors (control fibroblasts 1:1 *n* = 11, 1:3 *n* = 9, 1:9 *n* = 9, DOCK2-deficient fibroblasts 1:1 *n* = 5, 1:3 *n* = 5, 1:9 *n* = 5, *p < 0.05 unpaired two-tailed *t* test *p* = 0.0149, df = 18). **c** Wild-type (WT) iPS-MSPCs and *DOCK2*^*−/−*^*-*iPS-MSPCs inhibit T cell mitogenesis (iPS-MSPC WT 1:1 *n* = 4, 1:3 *n* = 4, 1:9 *n* = 4; *DOCK2* knockout iPS-MSPC 1:1 *n* = 8, 1:3 *n* = 8, 1:9 *n* = 8, unpaired two-tailed *t* test 1:3 *p* = 0.0090, df = 10; 1:9 *p* = 0.0059, df = 10); and (**d**) MLR in a dose-dependent manner (iPS-MSPC wild type 1:1 *n* = 4, 1:3 *n* = 4, 1:9 *n* = 4; *DOCK2* knockout iPS-MSPC 1:1 *n* = 6, 1:3 *n* = 6, 1:9 *n* = 6, unpaired two-tailed *t* test 1:1 *p* = 0.0398, df = 8; 1:3 *p* = 0.0016, df = 8, 1:9 *p* = 0.0029, df = 8). **e**–**h** Conditional siRNA-mediated DOCK2 knockdown in different stromal cells (see supplementary Fig. 10). **e**, **f** Healthy control fibroblasts or (**g**, **h**) iPS-MSPC, either MOCK transfected (gray bars) or transfected with DOCK2 siRNA 72 h prior to testing their immunomodulatory capacity inhibiting (**e**, **g**) T cell mitogenesis or (**f**, **h**) MLR. Mean values ± SD of T cell proliferation triplicates, percentage of inhibition after data normalization.

To assess whether DOCK2 functions, associated with T cell immunology, can also dictate immunomodulatory stromal cell functions, we tested the capacity of two *DOCK2-*mutated fibroblast lines from two SCID patients to modulate T cell proliferation (Fig. 1a II and Supplementary Fig. 9a). These fibroblast lines were selected as representing skin stromal cells. Compared to healthy donor fibroblasts, the *DOCK2-*mutated patient fibroblasts showed dose-dependent significant lack of inhibition of T cell mitogenesis (Fig. 3a) and allogeneic mixed lymphocyte reactions (Fig. 3b), respectively. More stringent direct comparison to autologous healthy fibroblasts (Fig. 1c) was hampered by donor variation of healthy control fibroblasts^24^.

We thus performed CRISPR/Cas9-mediated bi-allelic *DOCK2* knockout in healthy iPSCs to create *DOCK2-*deficient clones (PMUi002-A-1-3) directly comparable to their iPS-MSPC controls derived from the same initially picked iPSC clone (PMUi002-A) after differentiation (Supplementary Fig. 9b). This guided confirmation of our discovery of *DOCK2* expression regulating MSPC immunomodulatory capacity acquisition during mesodermal stromal differentiation (Fig. 1a III and Supplementary Fig. 9c). Direct head-to-head assessment confirmed dose-dependent significant lack of inhibition of mitogen-induced as well as allo-antigen-driven T cell proliferation by *DOCK2*^‒/‒^ iPS-MSPCs compared to unmutated control UCB-derived iPS-MSPCs (Fig. 3c, d). Furthermore, siRNA-mediated partial knockdown of *DOCK2* in control fibroblasts as well as iPS-MSPCs (Supplementary Fig. 10) 72 h in advance of adding the cells into the immunomodulation assay resulted in a significant dose-dependent reduction of the stromal immunomodulatory function (Fig. 3e–h).

**Fig. 4:**
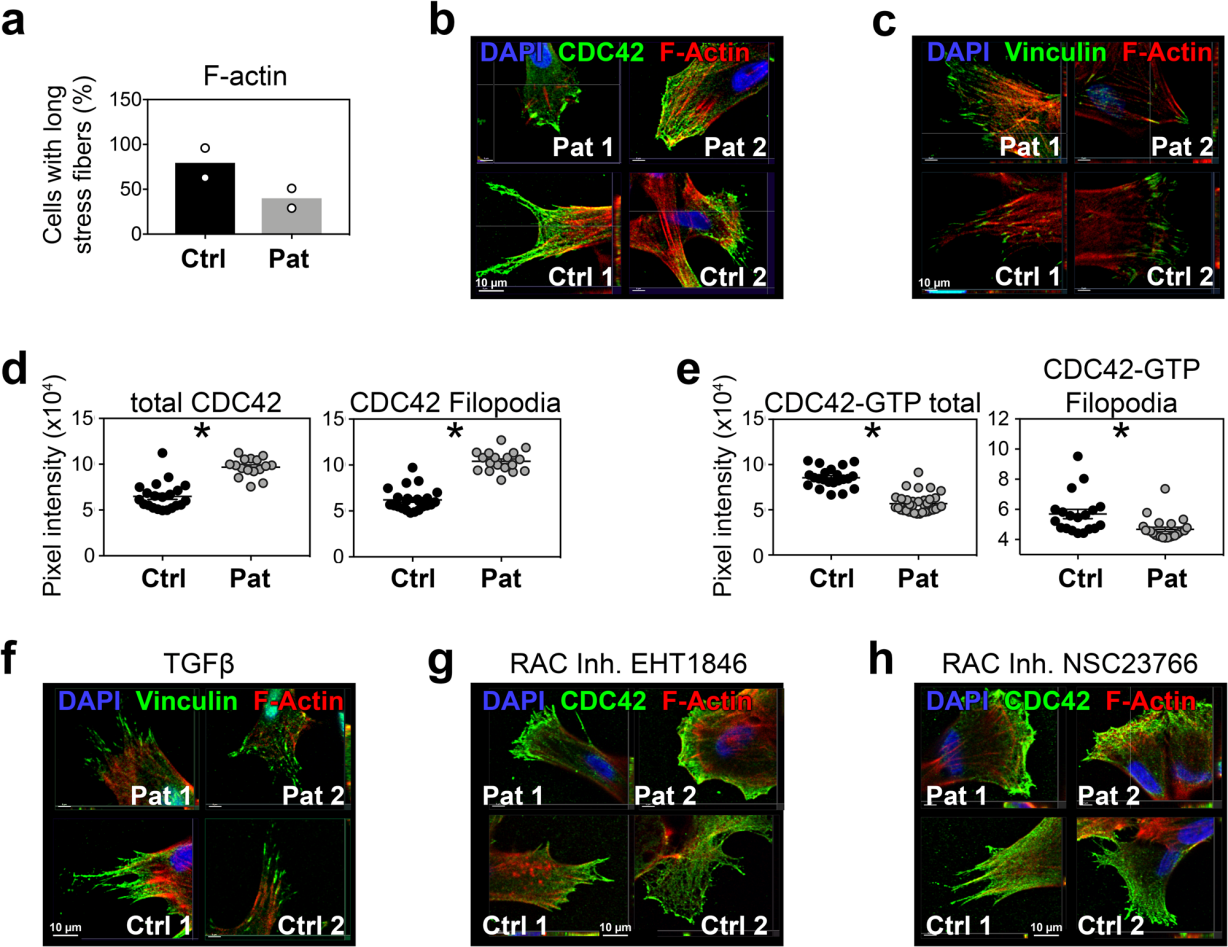
Subcellular localization and modulation of F-actin and cytoskeletal proteins in control fibroblasts and DOCK2-deficient patient fibroblasts. **a** Skin fibroblasts from controls (Ctrl., *n* = 2) and *DOCK2*-mutant patients (Pat., *n* = 2) were stained with phalloidin and the presence of long stress fibers enumerated. Representative immunofluorescence shown by reviewer demand in supplementary Fig. 12. **b** Confocal microscopy of Ctrl. And Pat. Fibroblasts, stained for CDC42 (green) and phalloidin (red); nuclear counterstain DAPI (blue) or (**c**) for vinculin (green), phalloidin (red) and DAPI (blue). **d** Enumeration of total of green CDC42 pixels in Ctrl fibroblasts (black circles) and *DOCK2*-mutant Pat. Fibroblasts in total cells (left graph, *n* = 21 control cells (obtained from 2 donors) and *n* = 16 patient cells (obtained from 2 donors), unpaired Mann–Whitney test, *p* < 0.0001) and filopodia (right graph, *n* = 23 control cells (obtained from 2 donors) and *n* = 18 patient cells (obtained from 2 donors), unpaired Mann–Whitney test, *p* < 0.0001). **e** Enumeration of the total of green CDC42-GTP pixels as in (**d**) for total cells (left graph, *n* = 35 control cells (obtained from 2 donors) and *n* = 54 patient cells (obtained from 2 donors), unpaired Mann–Whitney test, *p* < 0.0001) and filopodia (right graph, *n* = 19 control cells (obtained from 2 donors) and *n* = 24 patient cells (obtained from 2 donors), unpaired Mann–Whitney test, *p* = 0.0002). Confocal images of Ctrl or *DOCK2*-mutant Pat. fibroblasts treated for 60 min in vitro with (**f**) 250 ng/ml TGFbeta1 or 100 µM RAC1 inhibitors (**g**) EHT 1846 or (**h**) NSC23766 and stained for vinculin (green, **f**) or CDC42 (green, **g**, **h**), phalloidin (red) and DAPI (blue). Scale bars: 10 µm; statistics in (**d**) and (**e**) performed using Mann–Whitney *U* test.

DOCK2 is an activator of the small GTPases RAC and CDC42, both regulating cytoskeleton-dependent migratory responses in immune cells. These small GTPases also play an important role in secretory pathway, vesicle transport and autophagy^30^. In experiments studying consequences of *DOCK2* mutations in fibroblasts from SCID patients for cytoskeleton organization and formations of membrane protrusions, we found actin stress fibers and lamellipodia formed in both backgrounds, but CDC42- or vinculin-expressing filopodia formation reduced in *DOCK2*-mutant cells. The CDC42^+^ structures ended at the plasma membrane and did not protrude into filopodia (Fig. 4a–c). The total CDC42 amount appeared elevated, both in total, and in the filopodia. In line with an expected reduction in GEF activity due to the *DOCK2* mutations, we observed significantly reduced activated CDC42-GTP in the total cell and the filopodia of the patient fibroblasts (Fig. 4d, e). To validate this unexpected observation, we performed CDC42-GTP pulldown western blots. We found a significant mean 1.95-fold reduction of active CDC42-GTP in the patient fibroblasts compared to the control fibroblasts, and mean 3.87-fold CDC42-GTP reduction in the *DOCK2*^*‒/‒*^ iPSCs (0.71) compared to the wild-type iPSCs (2.77; Supplementary Fig. 11).

We next modulated DOCK2 GTPase activation in control and patient fibroblasts, showing that TGF-beta as GEF regulator^31^ reconstitutes filopodia formation and vinculin expression to the tips of the filopodia in *DOCK2*-mutant patient fibroblasts (Fig. 4f). In contrast, the RAC inhibitor EHT1846 had no effects and NSC23766 showed minimal reconstitution of CDC42 localization and filopodia compared to healthy fibroblasts (Fig. 4g, h).

## Discussion

Stromal cells are currently studied intensively in clinical trials to modulate immune responses^32^. Importantly, the underlying molecular mechanisms and the physiological role of a stromal immunomodulatory function are still obscure. Stromal cells form immune synapses which directly interact with T cells or indirectly modulate T cells via dendritic cells to inhibit immune responses^17,33^.

Our study of MSPC and iPS-MSPCp4/8 gene expression shows not only an enrichment of genes directly involved in regulating T cells but also of genes involved in small GTPase signaling, including *DOCK2* and *RAC2*. Rho GTPases are cytoskeletal regulators essential for cell morphology and motility, and guide vesicle-mediated processes including the secretory pathway and autophagy^34^. Adaptation of cells to environmental stressors is adjusted by complex signaling networks regulated by small GTPases CDC42, Rac, and Rho^35^. Two families of GEFs, MCF2 (better known as Dbl) and DOCK coordinate the spatio-temporal activation of RAC and CDC42 proteins and their signaling into the cell^36^. We identified the *DOCK2* gene as being upregulated during MSPC differentiation of iPSCs from both BM and UCB sources. DOCK2 has been considered as hematopoietic cell-specific GEF indispensable for chemotaxis and migration of T and B lymphocytes as well as hematopoietic stem cells^37,38^. Furthermore, *Dock2* was found to be upregulated and associated with increased CDC42 activation in repopulation-deficient hematopoietic stem cells^39^. In line with these results, it was reported that patients with inherited *DOCK2* mutations show severe immunodeficiency^28^. Both T and B cells from these patients not only showed defective chemotaxis, but also reduced RAC1 activation and actin polymerization. Our results clearly demonstrate *DOCK2* expression at high levels in different stromal cells and iPS-MSPCs. We did not find differences in migration of *DOCK2*^Δ/Δ^ compared to healthy fibroblasts in scratch assays (Supplementary Fig. 13). To determine the role of DOCK2 in MSPC-mediated suppression of T cell proliferation, we investigated fibroblasts from SCID patients with *DOCK2* mutations as well as iPS-MSPCs with deletion of *DOCK2*. This functional defect was associated with reduced CDC42 activation and defective formation of the lamellipodia-filopodia interface, resulting in shorter or absent filopodia. The filopodia formation was previously identified as a critical mechanism during MSPC-dendritic cell interaction during immune response initiation, where brief co-culture of MSPCs with dendritic cells impaired antigen-presentation by the latter^33^. Actin and actin disruption can also modulate the differentiation of stromal cells and their clonogenic progenitors, i.e. MSPCs. Indeed, in vitro disruption of F-actin with cytochalasin has been reported to reduce osteogenesis in a p38-dependent manner^40^. However, others report that cytochalasin D promotes osteogenesis, by stimulating recruitment of nuclear ARP2/3 to G actin^40^. Our own in vivo work suggests that, reduced F-actin may drive the loss of mature adipocytes over time^30^. Cell-intrinsic mechanisms through ubiquitin-like ISG15 could partly compensate for chronic loss of function of key Rho GTPase regulators such as *DOCK6*^34^.

Although the *DOCK2* gene was not found to be differentially methylated, other players in the regulation of GTPases were so. Among those, *EphB2* was one of the most upregulated and hypomethylated genes and was previously shown to be involved in T cell proliferation^41,42^. It was also shown that Dock2 and EphB2 play a role in lymphoid progenitor migration into the thymus^43,44^, and that EphB2 signaling activates CDC42-GEF Mcf2l^45^, suggesting EPHB2 signals through the same pathway as DOCK2.

DOCK2 is critically involved in the development of several inflammatory diseases, including allergy, graft rejection and even human immunodeficiency virus infection^46^. It was also discovered to mediate severe T and B cell immunodeficiency^28^. Our results using fibroblasts from two SCID patients with *DOCK2* mutations clearly demonstrate that reduced DOCK2 GEF function is not restricted to hematopoietic cells, but is also at the center of regulatory pathways in stromal immunosuppressive activity. Previously, immunomodulatory activity of stromal cells, including MSPCs, was mainly associated with soluble factors resulting from NFkB and JAH/STAT-dependent pathway activation^2,5,14^. The *DOCK2*^Δ/Δ^ SCID patient-derived fibroblast lines used in this study were previously shown to be particularly sensitive to virus-mediated cytolysis—a defect that could be partly ameliorated in the presence of interferon alpha 2a^28^. Here we also observed an increase of the immunomodulatory capacity of normal as well as patient fibroblasts in response to pro-inflammatory signaling (Supplementary Fig. 14). As data are lacking on the precise DOCK2 turnover kinetics we can just speculate that elevated GEF function co-regulates immunomodulatory function in healthy stromal cells. The fact that *DOCK2*^Δ/Δ^ SCID patient-derived fibroblasts also respond to interferons or other pro-inflammatory signals might rather argue in favor of additional DOCK2 GEF-independent signals co-regulating stromal cell instruction during immunomodulation. Considering immune synapse formation and cell–cell interaction, further experiments are required to determine if DOCK2 functions might also be involved in macrophage polarization.

DOCK2 as a GEF functions as an activator of CDC42. We found significantly reduced CDC42-GTP in pulldown assays in *DOCK2*^−/−^ compared to unmutated stromal cells. Activated CDC42-GTP drives condensation of the Arp2/3 complex required for F-actin polymerization, the actin-myosin network and filopodia formation. While the network is an important transporting vehicle for both endo- and exocytosis, the filopodia are important for synapse formation with interacting cells, such as T cells. Our results are consistent with the view that DOCK2 deficiency reduces CDC42 activation as well as the formation of F-actin filaments and filopodia. Considering the reduced immunomodulation by DOCK2-deficient stromal cells, we speculate that the stromal cells show either reduced endo- and exocytosis or reduced synapse formation with interacting cells, or both. Our study thus opens new avenues for understanding and manipulating the immune function of stromal cells in experimental clinical settings by targeting DOCK GEF and GPCR signaling.

## Methods

### Cell isolation, reprogramming and culture

Approval was obtained for human cell and tissue sample collection and genetic reprogramming from the Institutional Review Board (protocols 19–252, 18–243, 21–060, 19–284 and 415-E/1776/4-2014, Ethics Committee of the province of Salzburg). Adult samples were collected in accordance with the Declaration of Helsinki after written informed consent from healthy volunteers. Umbilical cord blood (UCB) samples were collected after written informed consent by the mother-to-be obtained prior to delivery of full-term pregnancies. MSPCs from bone marrow (BM) and UCB were isolated and expanded under animal serum-free conditions using pooled human platelet lysate (hPL) replacing fetal bovine serum and their purity, identity, and viability was characterized by flow cytometry as previously described^47–50^. Clonogenicity and differentiation capacity was assessed as previously described^51^. Peripheral blood mononuclear cells (PBMCs) were isolated by density centrifugation from random donor buffy coats as described^7^.

Induced pluripotent stem cells (iPSCs) were reprogrammed from primary MSPCs (derived from BM or UCB) by non-integrative Sendai Viral vector kit (CytoTune^TM^-iPS Sendai Reprogramming Kit encoding for Oct4, Sox2, KLF4 and c-Myc, Life Technologies Cat.No. A1378001) by the Harvard Stem Cell Institute (HSCI) iPS Core Facility (Cambridge, MA, USA). The reprogramming protocol was described by Fusaki and colleagues in 2009^52^ and adapted by the HSCI. Human iPSCs were characterized by cytogenetic analysis of at least twenty G-banded metaphase cells per clone and teratoma formation. The iPSC clones were registered at https://hpscreg.eu/ including detailed information.

Human iPSCs were initially transferred from mouse embryonic feeder layers to feeder-free conditions and thereafter maintained on a selected batch of Matrigel^®^ (Corning) in mTeSR^TM^1 (STEMCELL Technologies) medium. When reaching a cell density appropriate for splitting, iPSCs were harvested using Gentle Cell Dissociation Reagent (STEMCELL Technologies). Viable cells were counted and seeded at a density of 5 × 10^4^ cells per cm^2^ on Matrigel^®^ in mTeSR^TM^1 containing 10 µM Y-27632 ROCK pathway inhibitor (Selleckchem). After 24 h, the medium was changed to STEMdiff™ Mesoderm Induction Medium (STEMCELL Technologies) and the medium was replaced daily for the next consecutive four days. On day five, cells were harvested using TrypLE^TM^ (Thermo, Cat.12563-029) and the expression of early mesoderm markers brachyury and CD56 was determined by flow cytometry. Cells were seeded in EGM^TM^-2 containing hydrocortisone, human fibroblastic growth factor basic (hFGF-B), vascular endothelial cell growth factor (VEGF), recombinant insulin-like growth factor (R3-IGF), ascorbic acid, human epidermal growth factor (hEGF) (all Lonza, Basel, CH), preservative-free heparin 10 IU/mL (Biochrom), 5 mM N(2)-L-alanyl-L-glutamin (Dipeptiven®, Fresenius Kabi, Austria), 10% hPL and 10 µM Y-27632 at a cell density of 27,000 cells/cm^2^ without Matrigel® coating and cultured in humidified incubators (Binder CB210) at 37 °C and 5% CO_2_ in ambient air. Until passage five, iPS-MSPCs were passaged at a density of 27,000 cells/cm^2^ twice weekly with addition of Y-27632. For microscopical documentation EVOS XL (Thermo Fisher Scientific) was used. Differentiation assays were performed with iPSCs reprogrammed from MSPCs derived from BM (4 clones) or UCB (3 clones).

DOCK2-deficient patient cells: Biallelic mutations in *DOCK2* were identified and confirmed by Sanger sequencing in both patients. Patient 1 was homozygous for *DOCK2* dinucleotide insertions leading to frameshift and premature termination, Patient 2 was compound heterozygous for different missense and nonsense *DOCK2* mutations. Multiple sequence alignment showed that missense mutations affect evolutionarily conserved residues^28^. An additional request by one reviewer was to also create additional iPSCs from the existing patient fibroblasts. We did not fulfill this request in the review process because the generation of iPSCs would require new IRB votes to be submitted in the respective centers by the responsible transplant physician or pediatrician plus consent by the parents. As we are blinded to the identity of the cell donors, we are not aware of the fate of the children since transplantation. Furthermore, it is not clear whether we get access to the patient information required to contact the parents because legal regulation in most countries prohibits disclosure of patient (and donor) identity outside transplant registries.

### *DOCK2* knockout

For introduction of a knockout of the *DOCK2* gene (NCBI gene ID 1794), the web-based tool CRISPOR (http://crispor.tefor.net/) was used to identify possible guide sequences that target exon 37 of the *DOCK2* gene. Two guide sequences, for which CRISPOR calculated low off-target binding sites and high cutting efficiencies were selected for experimental evaluation (see Supplementary Table 3 for sequences). Subsequently, the selected guides were tested for their cutting efficiency and indel formation frequency by transfection of the iPSCs, sequencing of the target site and analysis using the TIDE algorithm (https://tide.deskgen.com/). Methodical details are described below. Application of guide-2 resulted in the higher cutting and indel formation frequency (69.4%) and was therefore used for the generation of the knockout clones.

For editing, the Alt-R CRISPR-Cas9 System (Integrated DNA technologies) was used. This included the following reagents: Alt-R® S.p. Cas9 Nuclease V3 (Cat. 1081058), Alt-R® CRISPR-Cas9 tracrRNA (Cat. 1073191) and crRNA (GAAGATCGCGGAGTTTGTAC). The gRNA duplex was generated by formation of *crRNA:tracrRNA duplex*. Briefly, 5 µL of the specific crRNA (100 µM) and 5 µL of tracrRNA (100 µM) were mixed and incubated for 5 min at 95 °C followed by down cooling to RT for 15 min. The RNP-complex was formed by mixing 2 µL of gRNA duplex and 2 µL of Alt-R® S.p. Cas9 Nuclease V3 (61 µM) and incubation for 35 min at RT.

Transfections were carried out using the P3 Primary Cell 4D-Nucleofector X Kit (Lonza, Cat. V4XP-3024) with program CM150 in the Lonza Nucleofector^TM^ device (Core Unit and X Unit). Briefly, iPSCs were harvested by incubation with TrypLE (Thermo, Cat.12563-029) to yield a single cell suspension. A total cell number of 1.3 ×10^6^ cells were resuspended in 100 µL of transfection buffer mix of the kit, 4 µL of the RNP-complex was added and transfected using program CM150 of the nucleofection device (Lonza). After transfection, cells were seeded in StemFlex^TM^ medium (Thermo Fisher, Cat. A3349401) supplemented with 10% CloneR (Stem Cell Technologies Cat. 05888) into one well of a Geltrex (Thermo Fisher Cat. A1413202) coated 6 well plate. Culture medium was changed daily with addition of CloneR for the first three days after transfection. On day four after transfection, the medium was switched to E8 medium^53^. To isolate clones, cells were passaged using TrypLE and seeded as single cells in Geltrex coated 6 well at a density of 50 - 300 cells/well. Individual iPSC colonies from these cultures were picked into the wells of a Geltrex coated 24 well plate using a pipet tip. After four days of culture cells were harvested from each well by 0.5 mM EDTA treatment. Half of the individual cell suspensions were frozen using Bambanker freezing medium (Nippon Genetics Europe, Cat. BB01-NP) and stored in liquid nitrogen and the other half was used to amplify the targeted genomic region using the Phire Animal Tissue Direct PCR Kit (Thermo Fisher, Cat. F-140WH) regarding the manufacturer’s instructions (Supplementary Table 3) for primer sequences.

PCR products were analyzed using 1.5% agarose gel electrophoresis to confirm amplification and allow subsequent clean up using the NucleoSpin Gel and PCR Clean-up Kit (Macherey Nagel, Cat. 740609.250). Samples were subjected to Sanger Sequencing (Microsynth Seqlab) and results were analyzed using Snapgene software (GSL Biotech LLC). Three clones were identified to carry a frameshift mutation resulting in a premature stop codon.

### *DOCK2* siRNA knockdown

We tested three specific siRNAs (Thermo Fisher Cat. 4392420; s4230-32) for *DOCK2* knockdown in healthy donor fibroblasts as well as iPS-MSPCs. Cells were grown in their standard growth medium described above, to 50% confluence, and transferred to Optimem medium (Thermo Fisher, Cat. Gibco™ 31985062) before adding Lipofectamine (RNAiMAX Reagent Thermo Fisher, Cat. 13778030) siRNA complexes according to manufacturer’s protocol. Transfected cells were harvested 72 h post transfection for checking knockdown efficacy by western blot, using a DOCK2-specific antibody (Thermo Fisher, Cat.MA5-26547) and for application in T cell proliferation assay.

### Flow cytometry immune phenotyping and T cell proliferation assay

Immune phenotyping of MSPCs, iPSCs and iPS-MSPCs was performed using a BD LSRFortessa™ (Becton Dickinson) and the following antibodies with their corresponding isotype controls: CD19-BUV395, CD29-APC, CD44-PE, CD45-APC, CD73-PE, CD90-BUV395, CD140a-BV421, CD140b-BV421, SSEA-4-PE, Tra-1-81-Alexa Fluor 647, HLA-ABC-BUV395 (BD), CD14-PE, CD31-eF450, CD34-PE-Cy7, CD56-PE, CD105-eF450, HLA-DR-eF450 (eBioscience), CD141-APC, CD146-PE-Vio770 (Milteny), brachyury-APC (R&D Systems) and Oct4-PE (Biolegend).

Dead cells were excluded based on FVD-eFluor^TM^520 (eBioscience^TM^) staining. For intracellular staining Fix & Perm solution (eBioscience^TM^) was used according to the manufacturer´s protocol. Repetitive analysis was performed (Oct4, *n* = 34; SSEA4, *n* = 32; Tra181, *n* = 67; brachyury, *n* = 29; CD140a, *n* = 14; CD140b, *n* = 26; MHC1, *n* = 11; CD90, *n* = 74; CD105, *n* = 78; CD29, *n* = 25; CD142, *n* = 25; CD56, *n* = 51; CD44, *n* = 29; CD146, *n* = 25; CD73, *n* = 73). Results were analyzed using Kaluza Analysis Software (Beckman Coulter).

Immunomodulatory potency of MSPCs, iPSCs and iPS-MSPCs was determined as described^7^. Briefly, peripheral blood mononuclear cells (PBMCs) from ten random donors were pooled, stained with carboxyfluorescein succinimidyl ester (CFSE, 2 μM, 15 min, 37 °C; Sigma) and cryopreserved in liquid nitrogen in order to have reference responders in multiple subsequent experiments. For immune modulation assays; 3 ×10^5^ CFSE pre-labeled PBMCs resuspended in RPMI-1640 supplemented with 10% hPL (for all assays performed with iPS-MSPCs) or AB serum (for all assays performed with *DOCK2* knockout cells), 2 IU/mL preservative-free heparin (Biochrom), 2 mM L-glutamine (Gibco), 10 mM HEPES (Gibco), 100 IU/mL penicillin and 100 μg/mL streptomycin (Sigma) were plated per well in triplicate in 96-well flat-bottomed plates (Corning). T cell proliferation in four-day mitogenesis assays was induced by 5 μg/mL phytohemagglutinin (PHA; Sigma). Allogeneic mixed leukocyte reactions due to the pooling of 10 independent PBMC donor-derived cells were measured at day seven. PBMC were cultured with or without graded numbers of MSPCs (250 μL total volume per well) in threefold serial dilution as indicated in the results section. All cultures were performed in humidified ambient air incubators (Binder CB210) at 37 °C and 5% CO_2_ in ambient air. Proliferation of viable CD3^+^ cells was analyzed using a Gallios 10-color flow cytometer and the Kaluza G1.0 software (both Coulter). Viable 7-aminoactinomycin-D-excluding (7-AAD; BD Pharmingen) CD3-APC^+^ (eBioscience) T cells were analyzed.

### RNASeq

RNA was isolated using the Macherey-Nagel RNA isolation kit according to the manufacturer’s protocol. The quality of total RNA isolates was analyzed using the Agilent RNA 6000 Nano Kit.

Poly-(A)-selection was performed utilizing the NEBNext Poly(A)mRNA Magnetic Isolation Module (NEB) according to the manufacturer’s requirements. mRNA libraries were prepared with the NEBNext Ultra RNA Library Prep Kit for Illumina (NEB). All libraries were analyzed with the Agilent DNA 1000 Kit and quantified using the Qubit® dsDNA BR Assay Kit (Thermo Fisher). After equimolar pooling all samples were sequenced on an Illumina HiSeq 1500 system with High Output chemistry v4 (50 cycles, single-read). Quality control was conducted using FASTQC (version 0.11.7—Bioinformatics Group at the Babraham Institute). Trimming and removal of residual adapters was done with AdapterRemoval^54^. Reads were then mapped to the Ensembl GRCh38 human genome using Tophat2 and Bowtie2. The number of mapped reads/gene (counts) was then calculated using HTseq. Genes were annotated using the Ensembl version 97. Expression values of protein coding genes were normalized using Deseq2 package in R (3.6.3). In order to see, how samples cluster together, a PC analysis of the total normalized dataset and hierarchical clustering using Euclidean distance were conducted. Differential expression analysis between the different groups was conducted using Deseq2. Genes with an adjusted *p*-value < 0.05 using Benjamini and Hochberg multiple testing correction^55^ were considered significantly differentially transcribed. Enrichment analysis (Go term enrichment, Gene set enrichment analysis, Kegg pathways and Panther pathways) were conducted using ‘clusterProfiler’ package in R. Benjamini and Hochberg multiple testing correction was used to adjust raw *p*-values for multiple testing (adj.*p*-value < 0.05 were considered significant).

### Methyl-Cap sequencing

For methylation pattern analysis, isolated DNA was sheared using a Bioruptor device (Diagenode). Subsequently, methylated DNA was isolated and eluted in high salt elution buffer using the MethylCap kit (Diagenode) according to the manufacturer´s instructions. Methylated DNA samples were prepared for next generation sequencing using the NEBNext ChIP-Seq Library Prep Master Mix Set for Illumina according to the manufacturer´s instructions. Amplified libraries were analyzed using the Bioanalyzer 2100 device and DNA 1000 Kit (Agilent) and concentrations were determined by means of Qubit 3.0 fluorometer device and the dsDNA BR assay (Thermo Fisher). Samples were sequenced using the Illumina HiSeq 1500 system (50 bp single reads) in three independent rounds. Sequence images were transformed to BCL files with the Illumina BaseCaller software and samples were demultiplexed to FASTQ files with bcl2fastq v2.17.1.14. Same procedures as above were conducted for quality control, read trimming and adaptor removal. Reads were then mapped to GRCh38 human genome using Bowtie2. Differentially methylated regions were identified using QSEA R package^56^.

### Scratch assay

For wound repair studies, fibroblasts were seeded at cell density of 20,000 cells per well in 24 well plates and cultured until confluence (around 24 h). Cells were serum starved overnight in 0.2% hPL. After standardized scratch of the confluent layer with a 200 µL pipette tip, medium was refreshed and cultures were introduced into a Okolab incubator system surrounding a Nicon Eclipse Ti. Cell movement was monitored by acquiring video sequences using NIS-Elements software covering a time period of 12 h. The area of wound repair was determined using TScratch software^57^ (Supplementary Fig. 13).

### Immunofluorescence, Rac inhibition, CDC42-GTP pulldown

We seeded cells on collagen-coated glass coverslips. Adhered cells were fixed with 4% PFA for 15 min at room temperature, then washed in PBS and permeabilized for 10 min in 0.1% TritonX100/PBS. After 30 min blocking in 10% FBS/Dako wash buffer, anti-CDC42 antibody (Abcam, ab187643), anti-CDC42-GTP antibody (active CDC42, NewEastBioscience 26905) or the corresponding isotype control (Abcam ab172730) were applied 1:500 and incubated overnight at 4 °C. After washing in Dako wash buffer, the phalloidin Alexa Flour 568 (1:40, Invitrogen) and the goat-anti-rabbit-Alexa Fluor 488 secondary antibody (1:500, Invitrogen) were applied in 10% FBS/Dako wash buffer for 1 h at RT. Finally, we washed the coverslips in Dako buffer and mounted them on glass slides, in RotiMount FluorCare (Roth) including DAPI. Confocal pictures were taken using a Zeiss LSM 710 and ZEN software. Rac inhibition was performed for 1 h at 37 °C for both inhibitors (EHT1864 and NSC23766). TGFbeta1 treatment was performed for 1 h at 37 °C. For immunofluorescence quantification pictures were taken under standardized conditions (light intensity, exposure, diaphragm) as described previously^30^. We determined total CDC42 and active CDC42-GTP content by assessing total pixels number per cell using ImageJ software (v1.52). For assessing stress fibers, each microscopic field was counted for a) the total number of cells; b) the number of cells that display long stress fibers; c) the number of cells that mainly showed short or irregular fiber formation (intermediate) or no formation of stress fibers. Active CDC42-GTP pulldown Western blots were done using a detection kit following manufacturer’s instruction (#16119; Thermo Fisher).

### Statistics and reproducibility

Statistical analysis of the results was performed using One-Way ANOVA analysis of variance with a confidence interval of 95% and corrected for multiple comparisons using the Holm Sidak algorithm in GraphPad Prism version 7.03. A *p*-value of < 0.05 was defined as significant. For the *DOCK2-*mutant patient fibroblasts, we got cell lines from two patients. We used a minimum of three biological replicates for all experiments in combination with technical replication.

### Reporting summary

Further information on research design is available in the Nature Research Reporting Summary linked to this article.

## Supplementary information

Peer Review File

Supplementary Information

Description of Additional Supplementary Files

Supplementary Data 1

Reporting Summary

## Data Availability

All fastq and raw counts files for RNA-seq and MethylCap-seq were uploaded to gene expression omnibus database: GSE189898 or GSE189895 (only RNA-seq) or GSE189896 (only MethylCap-seq). Chart plots raw numbers are provided in Supplementary Data 1. Uncropped Western Blot pictures are provided in Supplementary Fig. 15 (in Supplementary Information file). The iPSC lines used in this manuscript were all registered in hPSCreg (https://hpscreg.eu)

## Code availability

Bash and R scripts used for RNA-seq and MethylCAP-seq analysis were uploaded on Zenodo (10.5281/zenodo.7113105)
