# Supplementary material for: Extra-hematopoietic immunomodulatory role of the guanine-exchange factor DOCK2
Source: Commun Biol. 2022 Nov 15;5:1246. doi: 10.1038/s42003-022-04078-1 (PMC9666545; doi:10.1038/s42003-022-04078-1)
Supplement: Supplementary file 2 — Supplementary Information [file 42003_2022_4078_MOESM2_ESM.pdf]

Extra-hematopoietic immunomodulatory role of the guanine exchange factor DOCK2

Cornelia Scharler[1], Rodolphe Poupardin[1], Patricia Ebner-Peking[1], Martin Wolf[1], Christina Schreck[2], Gabriele Brachtl[1], Andre Cronemberger Andrade[1], Linda Krisch[1,4], Laurence Daheron[3], Katharina Schallmoser[4], Karsten Jürchott[5], Judit Küchler[5], Harald Stachelscheid[5], Hans-Dieter Volk[5], Robert AJ Oostendorp[2], Dirk Strunk[1]

[1]Cell Therapy Institute, Spinal Cord Injury and Tissue Regeneration Center, Paracelsus Medical University (PMU), Salzburg, Austria

[2]Technical University of Munich, School of Medicine, Internal Medicine III, Munich, Germany

[3]HSCI iPS Core Facility, Harvard University, Cambridge, USA

[4]Department of Transfusion Medicine and SCI-TReCS, PMU, Salzburg, Austria

[5]BCRT & Institute of Medical Immunology, Charité - Universitätsmedizin Berlin, Germany

DATA SUPPLEMENT

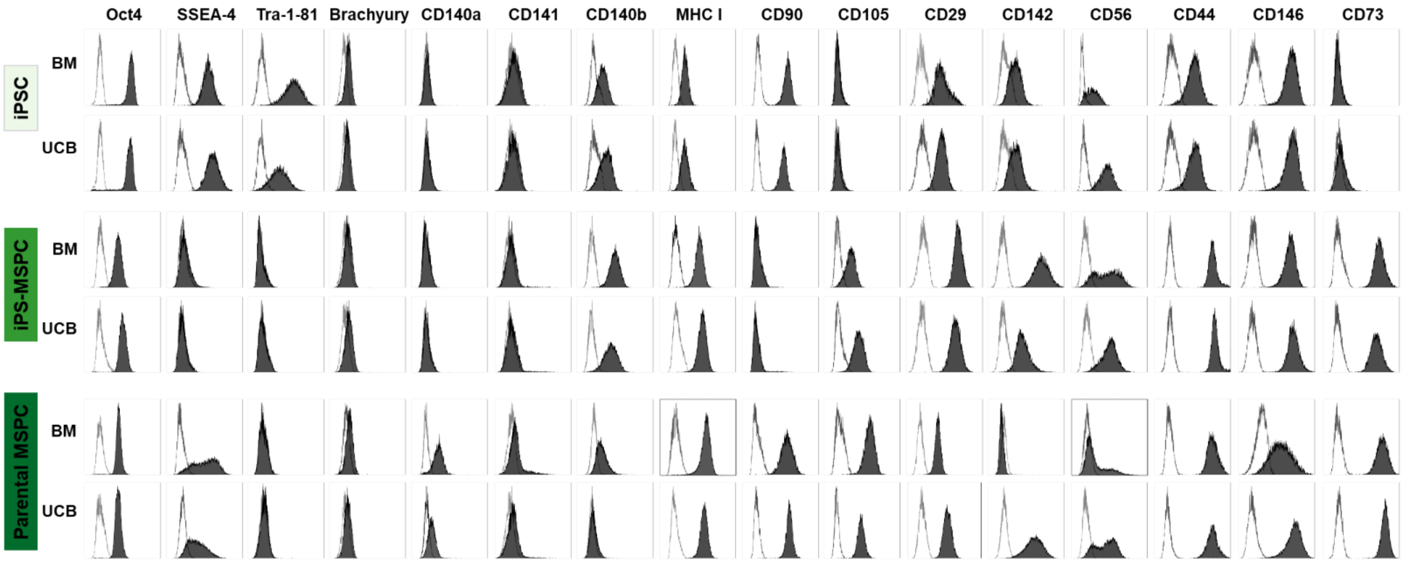

Supplementary Fig. 1: Marker expression profile during differentiation of iPSCs into iPS-MSPCs compared to parental BM and UCB MSPCs. Representative histograms of marker expression by iPSCs and iPS-MSPCs p8 compared to parental MSPCs as indicated. Histograms show fluorescent cell surface staining by fluorescent monoclonal antibodies as indicated (grey shading) and corresponding isotype controls (open histograms). Histograms show the populations following a hierarchical gating strategy: size and granularity, doublet exclusion, live cell population and corresponding marker identification.

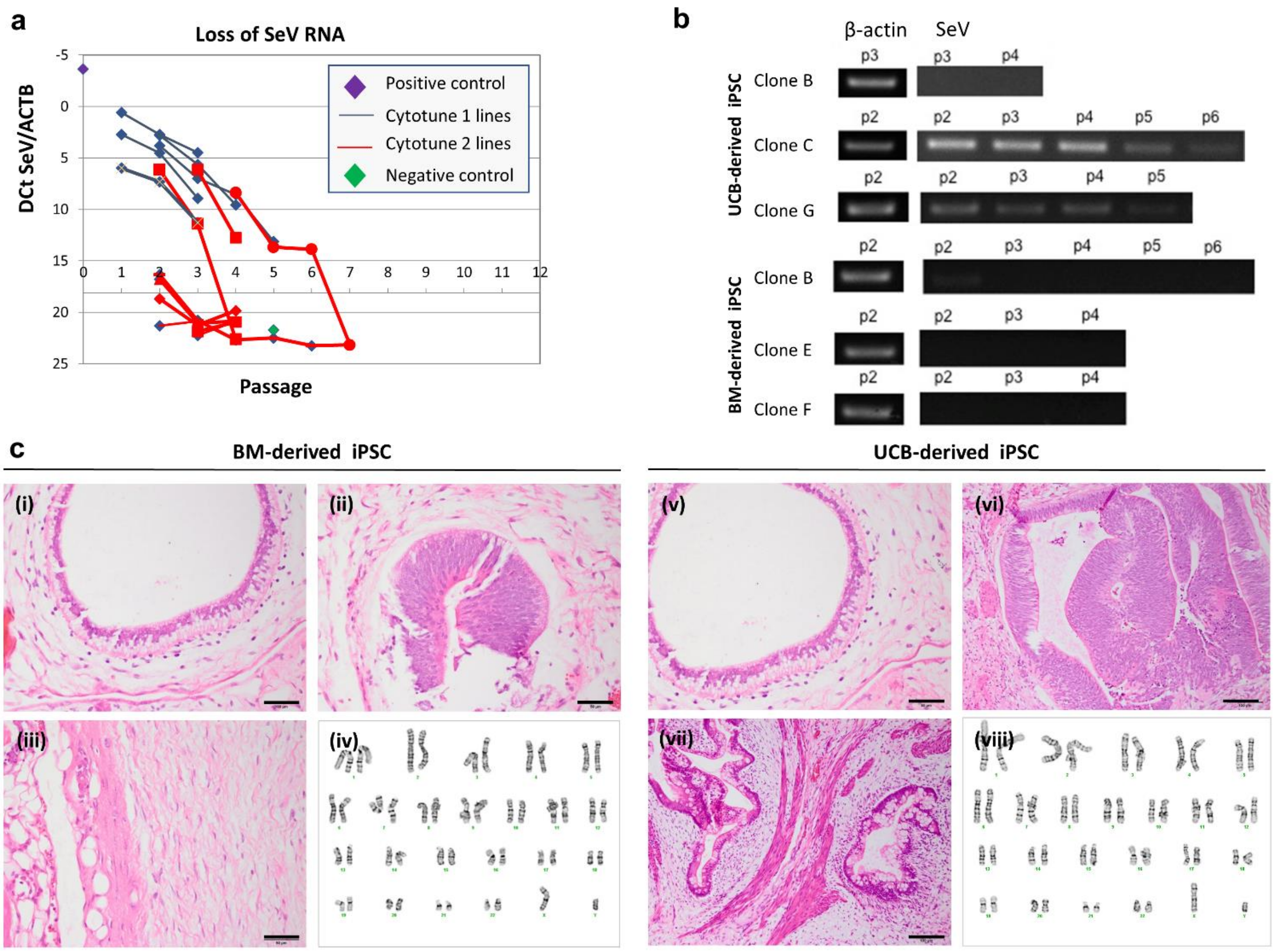

Supplementary Fig. 2: Characterization of iPSC lines. (**a**) Sendai Virus (SeV) RNA elimination testing comparing reprogramming with two different cytotune reprogramming reagents (1, 2). (**b**) Representative PCR results of SeV elimination, compared to b-actin control, for representative individual iPSC clones PMUi001B and PMUi002C. (**c**) Teratoma assay of BM-derived and UCB-derived iPSCs. Hematoxylin and eosin staining showing (**i**, **v**) endoderm (**ii**, **vi**) ectoderm (**iii**, **vii**) mesoderm structures in histology after injection of iPSCs from BM- and UCB-derived MSPCs as indicated, confirming pluripotency in representative teratoma formation tests. (**iv, viii**) G banding showing a normal karyotype of representative BM- and UCB-derived iPSCs as indicated. Scale bars: (i, vii) = 100 µm; (all others) = 50 µm.

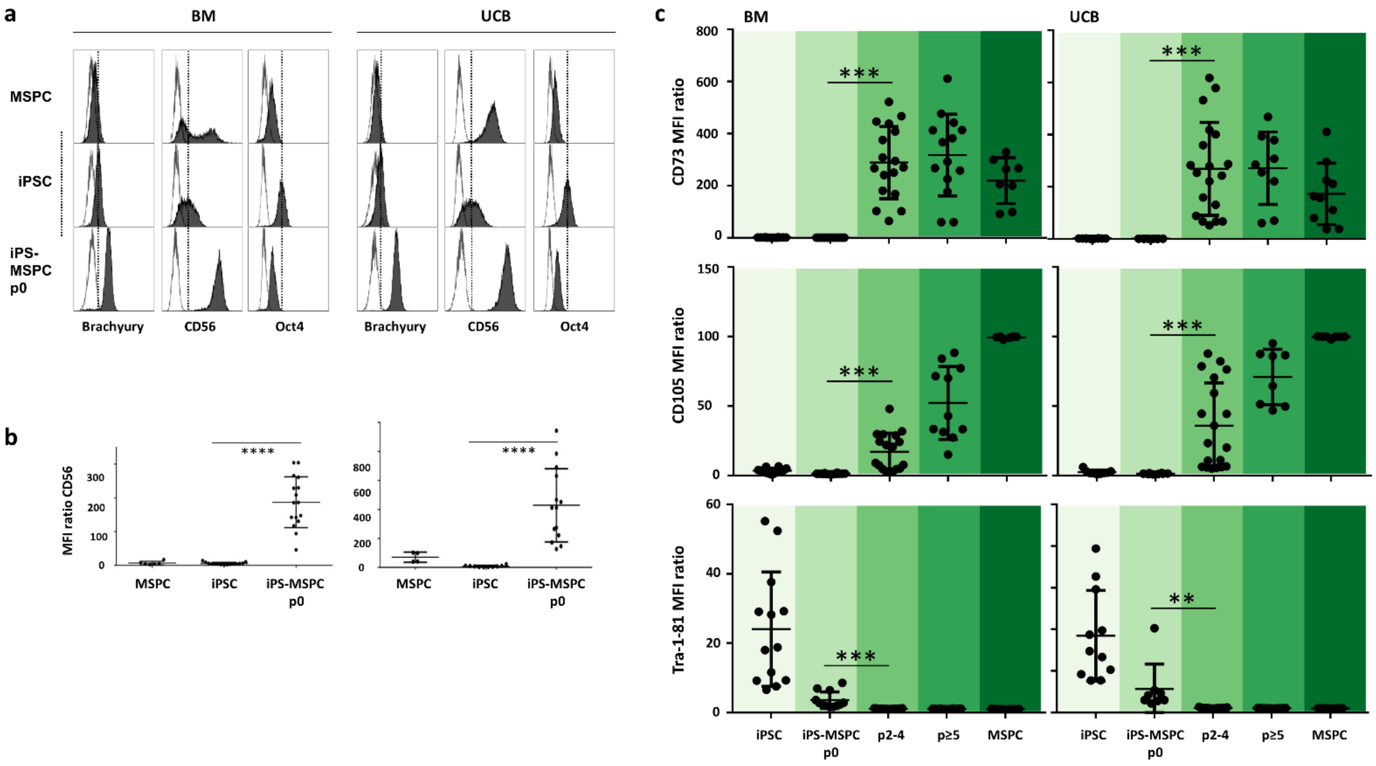

Supplementary Fig. 3: Mesoderm induction and stromal marker monitoring of iPS-MSPCs. (**a**) Representative flow cytometry histograms of parental BM- and UCB-MSPCs, their iPSC progeny and the respective mesoderm induced iPS-MSPCs at p0 are depicted. Cell surface anti-CD56 and intracellular anti-brachyury and anti-Oct4 fluorescent staining (grey), as histogram overlay with corresponding isotype controls (open histograms). Dashed lines indicate mean fluorescence of marker expression on/in iPSCs for comparison; one representative staining panel. Histograms show the populations obtained following hierarchical gating strategy: size and granularity, doublet exclusion, live cell population and corresponding marker display. (**b**) Quantification of CD56 expression (MFI ratio) to monitor reproducible and significant upregulation during induction of mesoderm in iPS-MSPCs at p0 (unpaired two-sided t-test with Welch´s correction, **** $p < 0.0001$, BM: df = 15.06, MSPC n = 6, iPSC n = 15, iPS-MSPC p0 n = 16; UCB: df = 13.02, MSPC n = 4, iPSC n = 10, iPS-MSPC p0 n = 14). (**c**) MSPC markers CD73 and CD105 and the pluripotency marker Tra-1-81 by flow cytometry monitoring in the course of MSPC-to-iPSC-to-iPS-MSPC development as indicated (n = 6 - 19). Green colour code corresponding to Fig.1 & 2. (c) Shown are MFI ratios of surface markers after flow cytometric analysis CD73, CD105 and Tra-1-81. Statistics were perfomed by unpaired two-tailed t test with Welch´s correction, CD73: BM $p < 0.0001$, df = 16.00, iPS-MSPC p2 - 4 n = 11, iPS-MSPC p0 n = 17; UCB: $p < 0.0001$, df = 18.00, iPS-MSPC p2 - 4 n = 8, iPS-MSPC p0 n = 19; CD105: BM $p < 0.0001$, df = 10.00, iPS-MSPC p > 5 n = 12, iPS-MSPC p0 n = 11; UCB: $p < 0.0001$, df = 7.004, iPS-MSPC p > 5 n = 8, iPS-MSPC p0 n = 8; Tra-1-81 BM $p = 0.0007$, df = 12.53, iPS-MSPC p0 n = 13, iPSC n = 12; UCB $p = 0.0048$, df = 16.07, iPS-MSPC p0 n = 11, iPSC n = 8; Error bars represent standard deviation.

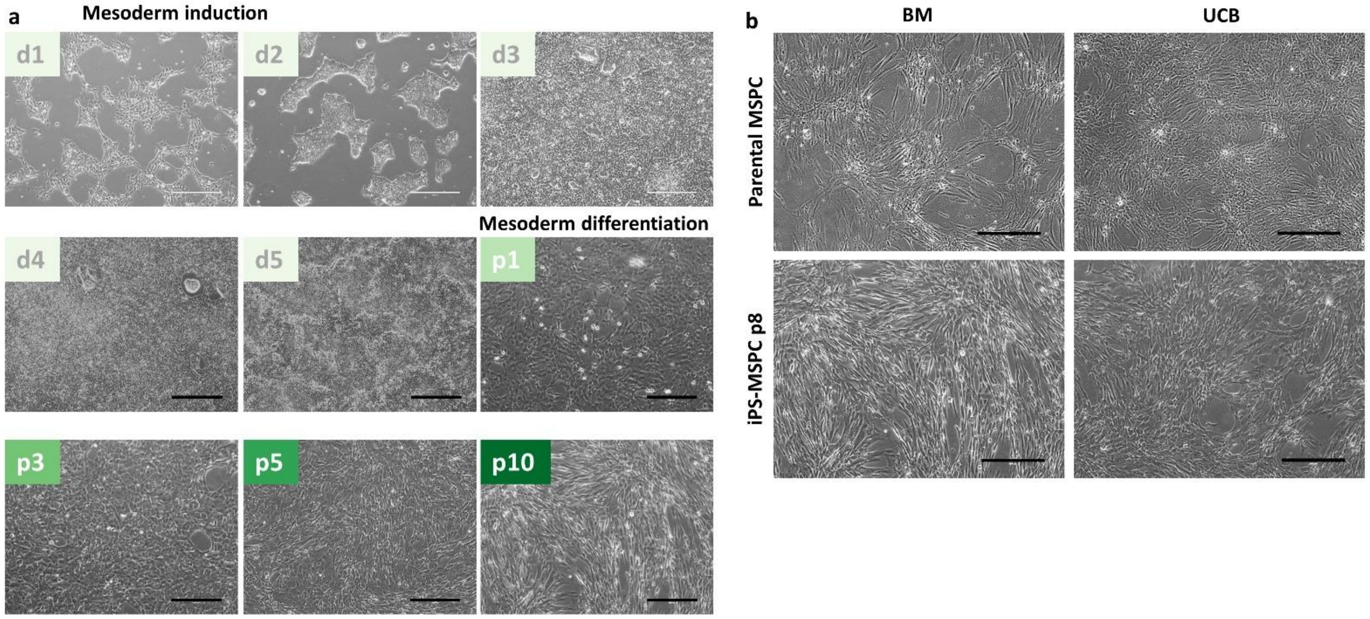

Supplementary Fig. 4: Mesoderm induction and iPS-MSPC differentiation. (**a**) Cells were monitored daily by phase contrast microscopy after medium change. P0 representing initial mesoderm induction culture (d1-5) before first passage (p1). Representative pictures of BM-MSPC-derived iPSCs & iPS-MSPC progeny. (**b**) Morphology of BM and UCB-derived parental MSPCs compared to their iPS-MSPC progeny. Representative pictures shown (scale bars 400 µm).

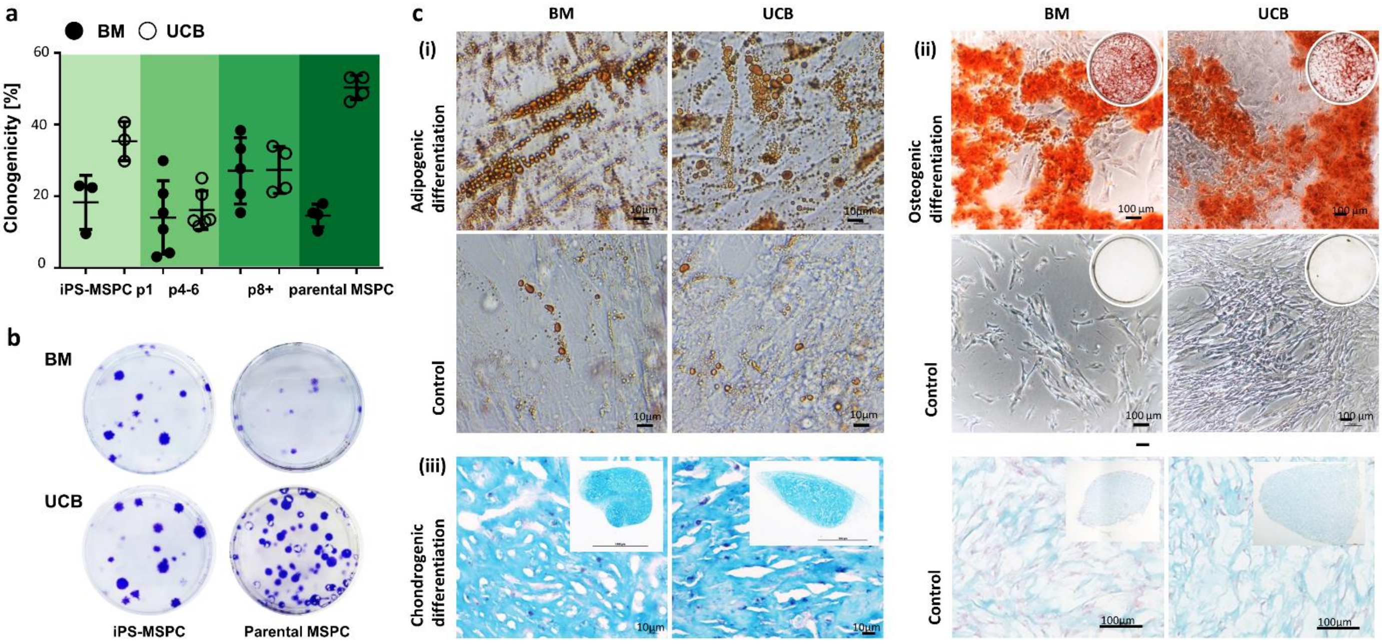

Supplementary Fig. 5: Clonogenicity and differentiation potential of iPS-MSPCs. (**a**) Colony-forming units of fibroblasts (CFU-F) capacity of BM- vs. UCB-derived iPS-MSPCs compared to their respective parental MSPCs was assessed at a density of 3 cells per cm^2 in the presence of ROCK inhibitor at seeding, corresponding to the differentiation protocol, for cells at passage 1 to 5. Primary MSPCs and iPS-MSPCs passage ≥ 8 were seeded without ROCK inhibitor– (n = 3 - 6). Error bars represent standard deviation. (**b**) Representative crystal violet stained CFU-F colony assay plates shown. (**c**) Adipogenic and osteogenic differentiation potential of iPS-MSPCs differentiated from BM- or UCB-derived iPSCs was revealed by positive Oil Red O (i) and Alizarin S Red (ii) staining, respectively, compared to negative control

cultures. Chondrogenic differentiation was assessed by Alcian blue staining accordingly compared to negative control (iii).

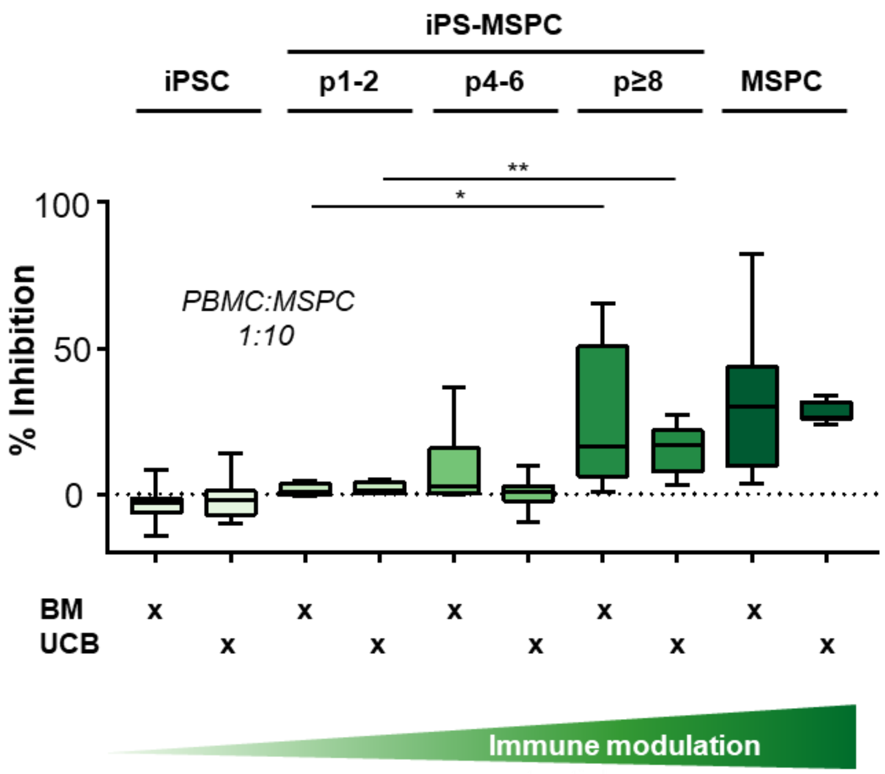

Supplementary Fig. 6: Dose dependent inhibition of T cell mitogenesis by iPS-MSPCs (1:9 ratio).

Corresponding to Fig.1c BM: iPSC n = 10, iPS-MSPC p1-2 n = 6, iPS-MSPC p4-6 n = 8, iPS-MSPC p≥8=7, MSPC n = 9; UCB: iPSC n = 7, iPS-MSPC p1-2 n = 5, iPS-MSPC p4-6 n = 8, iPS-MSPC p≥8 n = 7, MSPC n = 7 BM, n = 6-10; UCB, n = 5-8 (unpaired two-tailed t test BM p = 0.0433, df = 11; UCB p = 0.0087, df = 10). Error bars represent standard deviation.

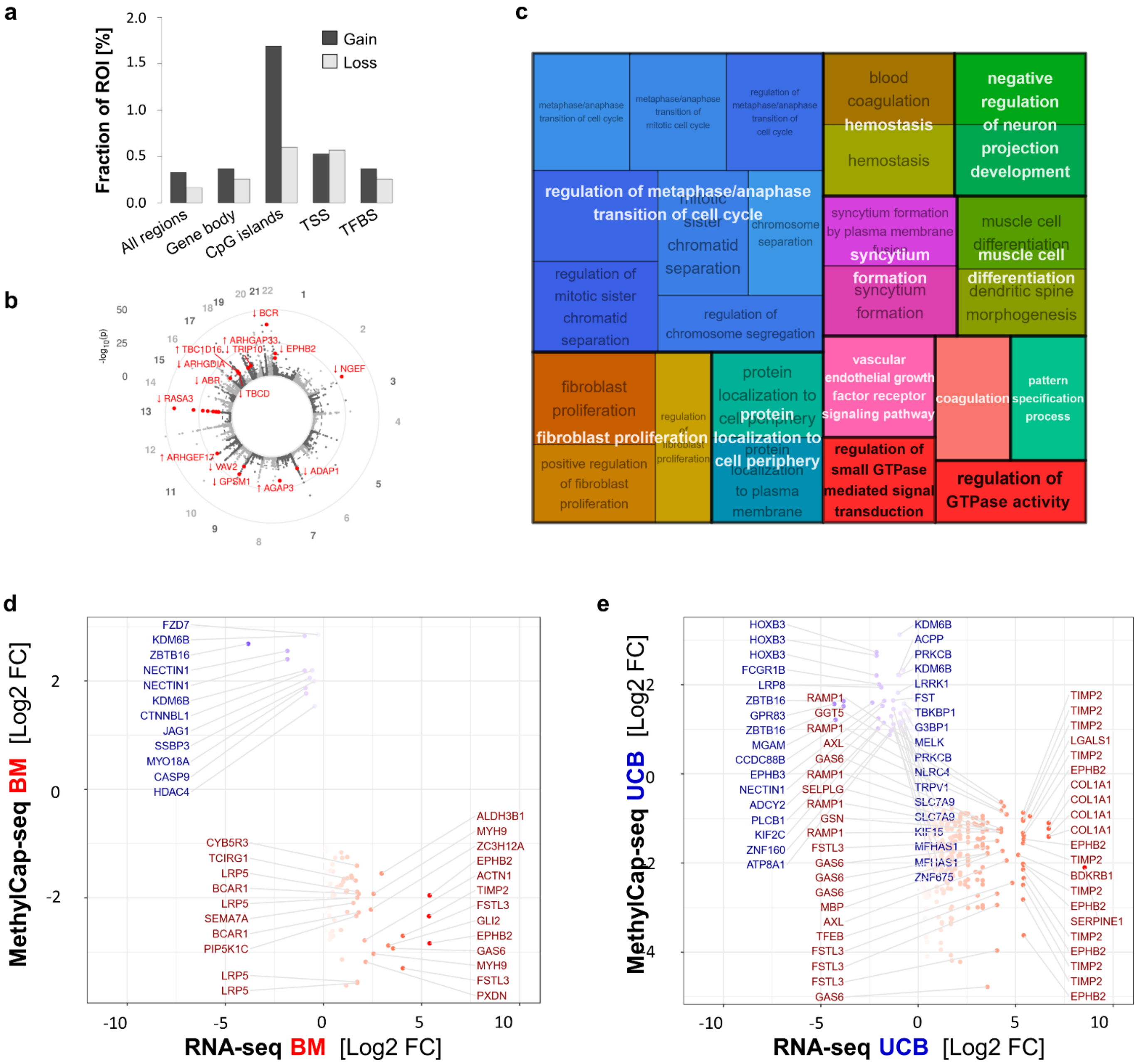

Supplementary Fig. 7: Methylome analysis during MSPC-to-iPS-to-MSPC ontogeny. (**a**) Feature enrichment showing the percentage of regions of interest (ROI; all regions, gene body, CpG islands, transcription starting sites (TSS) and transcription factor binding sites (TFBS) which gained or lost methylation between iPS-MSPCs p8 and p0. (**b**) Circular Manhattan blot illustrating the differentially methylated regions between iPS-MSPCs p8 and p0 on the whole genome with chromosome numbers on the external ring. Each dot corresponds to a differentially methylated region (500 base pair regions). The height of the dot correlates with the level of significance ($-\log_{10}$ adjusted p value, Benjamini-Hochberg correction). Highly significant windows overlapping genes belonging to the GO terms 'regulation of GTPase activity' and 'regulation of small GTPase mediated signal transduction' were highlighted in red. Arrows before gene names indicates whether genes are hypo- (downwards arrow) or hypermethylated (Upwards arrows). (**c**) Gene enrichment (using Biological Process terms) conducted on the genes found highly significantly differentially methylated ($-\log_{10}$ adjusted p value > 10 or adjusted p value < 1×10^{-10}) in the Manhattan plot (Fig 7b). **(d,e)** Genes involved in immune system process found significantly (adjusted p value < 0.05) overexpressed (RNAseq) and hypomethylated (MethylCap-seq) or underexpressed and hypermethylated, respectively, are shown with red dots or blue dots, for (**c**) BM and (**d**) UCB, p8 vs. p0, respectively.

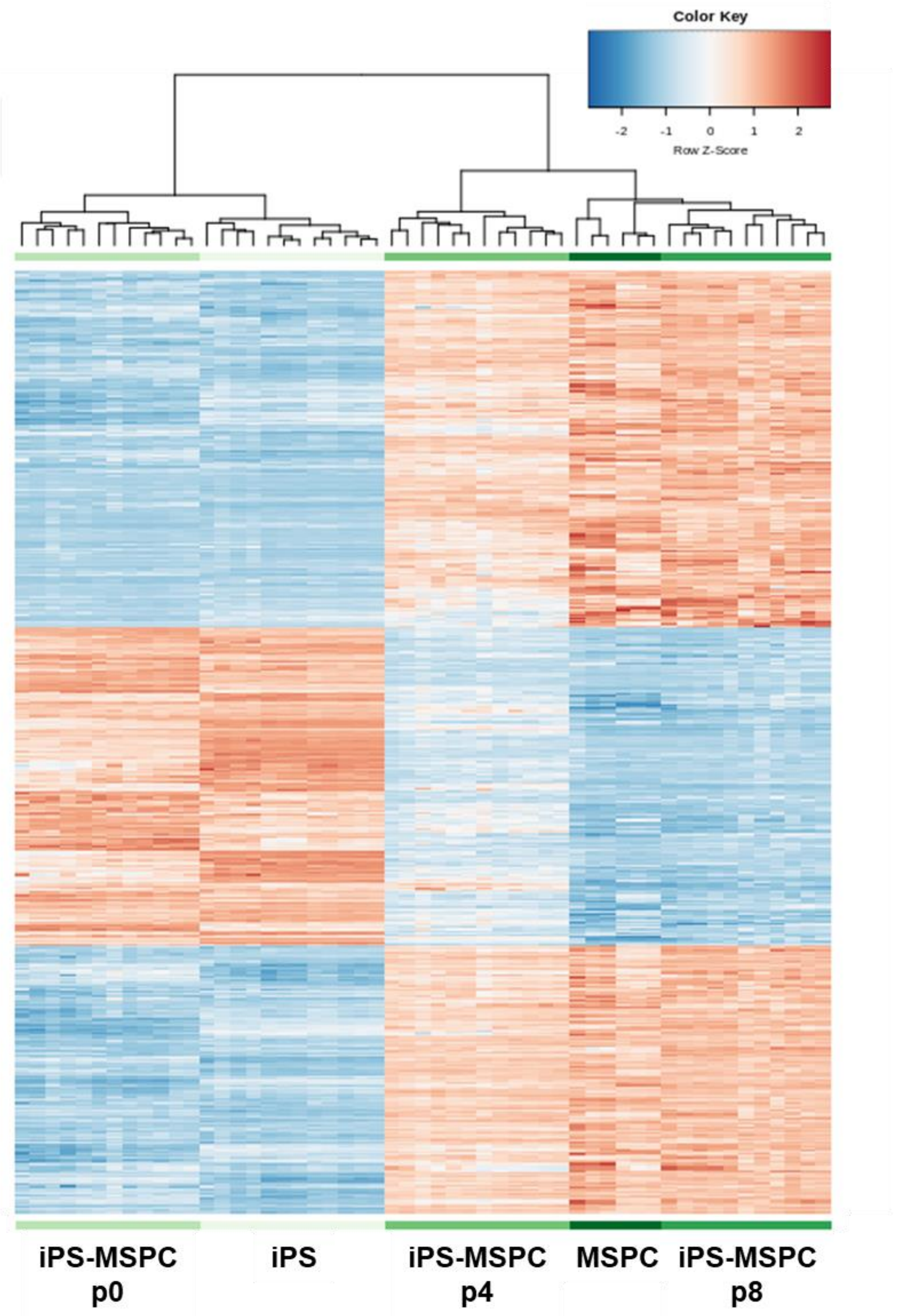

Supplementary Fig. 8a: Hierarchical clustering heat map with expression values of the 500 most variable genes.

Genes shown in rows while the different samples are column listed. Side color bar on the top and bottom indicates the different cells or passages. Gene expression values were row Z-score normalized where lower expression is denoted by blue and higher expression by red color as shown in the legend above iPSCs (n = 12), p0 iPS-MSPCs (n = 12), p4 iPS-MSPCs (n = 12), p8 iPS-MSPCs (n = 11), MSPCs (n = 6).

Supplementary Fig. 8b (next page)**: Top 200 upregulated in mature iPS-MSPCs and parental MSPCs.** Hierarchical clustering heat map with expression values of 200 most upregulated genes within the GO category 'immune system process' in parental MSPCs vs p0 that were also found upregulated in iPS-MSPC p8 vs iPS-MSPC p0. Genes are shown in rows, different samples in columns. Side color bar on the top indicates the different cells or passages. Gene expression values were row Z-score normalized. Lower expression denoted by blue and higher expression by red color. iPSCs (n = 12), p0 iPS-MSPCs (n = 12), p4 iPS-MSPCs (n = 12), p8 iPS-MSPCs (n = 11), MSPCs (n = 6). Genes belonging to T-cell proliferation marked **blue** and GPCR signaling in **red**.

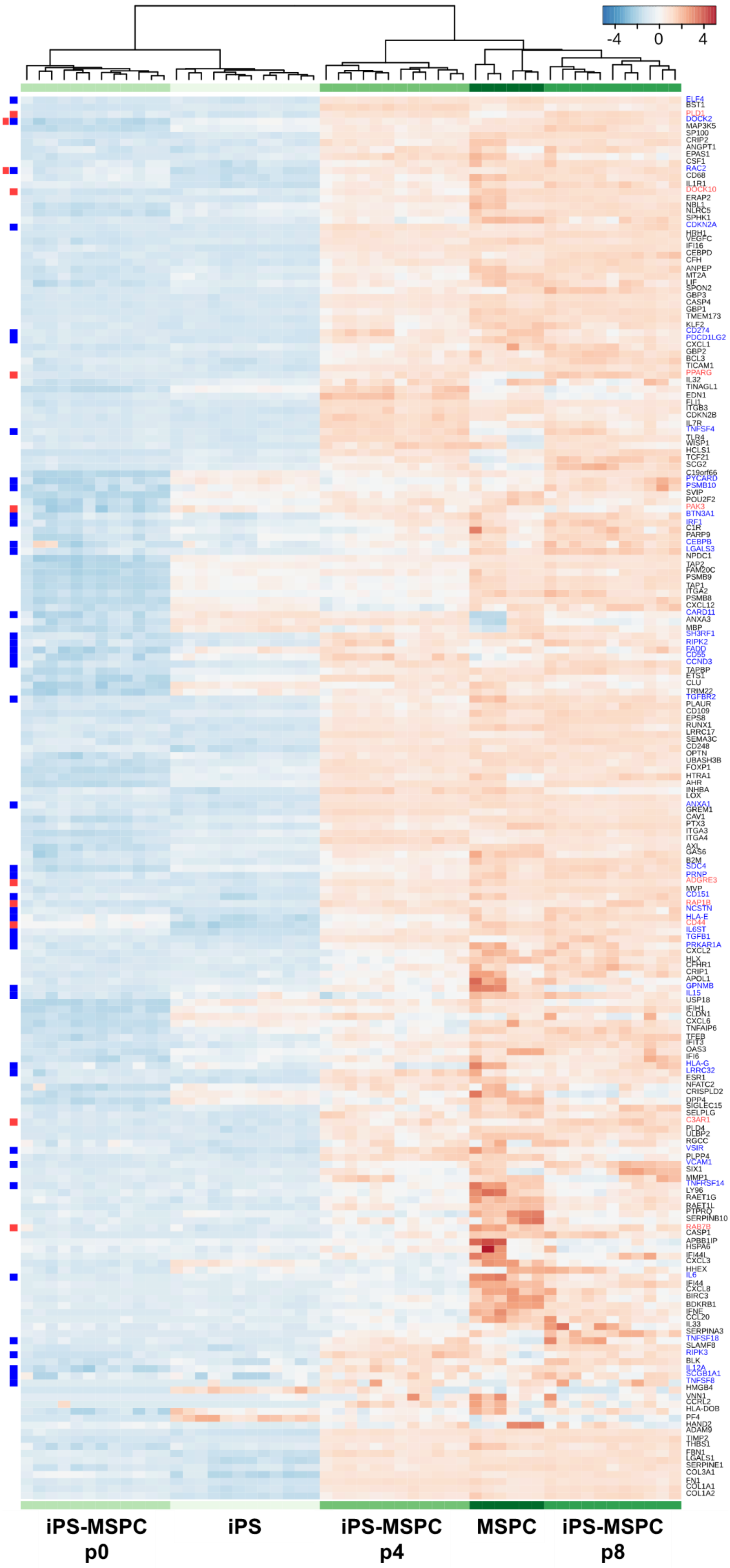

-4
0
4
ELF4
BST1
PLD1
DOCK2
MAP3K5
SP100
CRIP2
ANGPT1
EPAS1
CSF1
RAC2
CD68
IL1R1
DOCK10
ERAP2
NBL1
NLRC5
SPHK1
CDKN2A
HRH1
VEGFC
IFI16
CEBPD
CFH
ANPEP
MT2A
LIF
SPON2
GBP3
CASP4
GBP1
TMEM173
KLF2
CD274
PDCD1LG2
CXCL1
GBP2
BCL3
TICAM1
PPARG
IL32
TINAGL1
EDN1
FLI1
ITGB3
CDKN2B
IL7R
TNFSF4
TLR4
WISP1
HCLS1
TCF21
SCG2
C19orf66
PYCARD
PSMB10
SVIP
POU2F2
PAK3
BTN3A1
IRF1
C1R
PARP9
CEBPB
LGALS3
NPDC1
TAP2
FAM20C
PSMB9
TAP1
ITGA2
PSMB8
CXCL12
CARD11
ANXA3
MBP
SH3RF1
RIPK2
FADD
CD55
CCND3
TAPBP
ETS1
CLU
TRIM22
TGFBR2
PLAUR
CD109
EPS8
RUNX1
LRRC17
SEMA3C
CD248
OPTN
UBASH3B
FOXP1
HTRA1
AHR
INHBA
LOX
ANXA1
GREM1
CAV1
PTX3
ITGA3
ITGA4
AXL
GAS6
B2M
SDC4
PRNP
ADGRE3
MVP
CD151
RAP1B
NCSTN
HLA-E
CD44
IL6ST
TGFB1
PRKAR1A
CXCL2
HLX
CFHR1
CRIP1
APOL1
GPNMB
IL15
USP18
IFIH1
CLDN1
CXCL6
TNFAIP6
TFEB
IFIT3
OAS3
IFI6
HLA-G
LRRC32
ESR1
NFATC2
CRISPLD2
DPP4
SIGLEC15
SELPLG
C3AR1
PLD4
ULBP2
RGCC
VSIR
PLPP4
VCAM1
SIX1
MMP1
TNFRSF14
LY96
RAET1G
RAET1L
PTPRO
SERPINB10
RAB7B
CASP1
APBB1IP
HSPA6
IFI44L
CXCL3
HHEX
IL6
IFI44
CXCL8
BIRC3
BDKRB1
IFNE
CCL20
IL33
SERPINA3
TNFSF18
SLAMF8
RIPK3
BLK
IL12A
SCGB1A1
TNFSF8
HMGB4
VNN1
CCRL2
HLA-DOB
PF4
HAND2
ADAM9
TIMP2
THBS1
FBN1
LGALS1
SERPINE1
COL3A1
FN1
COL1A1
COL1A2
iPS-MSPC p0
iPS
iPS-MSPC p4
MSPC
iPS-MSPC p8

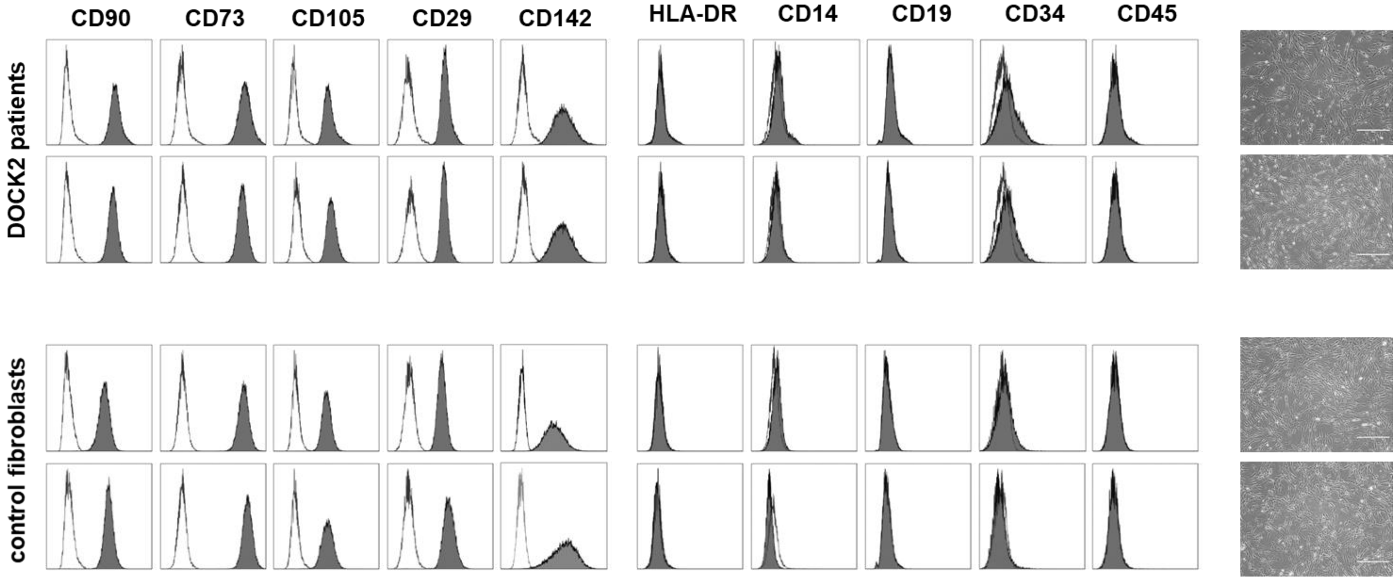

Supplementary Fig. 9a: Immune phenotyping of fibroblasts derived from DOCK2 patients. Representative histograms show fluorescent cell surface staining intensity of anti-CD90, anti-CD73, anti-CD105, anti-CD142, anti-CD29, anti-HLA-DR, anti-CD14, anti-CD19, anti-CD34 and anti-CD45 monoclonal antibodies conjugated to fluorophores (gray shading) and corresponding isotype control (no shading). Representative morphology of DOCK2 patients and control fibroblasts by phase contrast microscopy, scale bar = 400 µm.

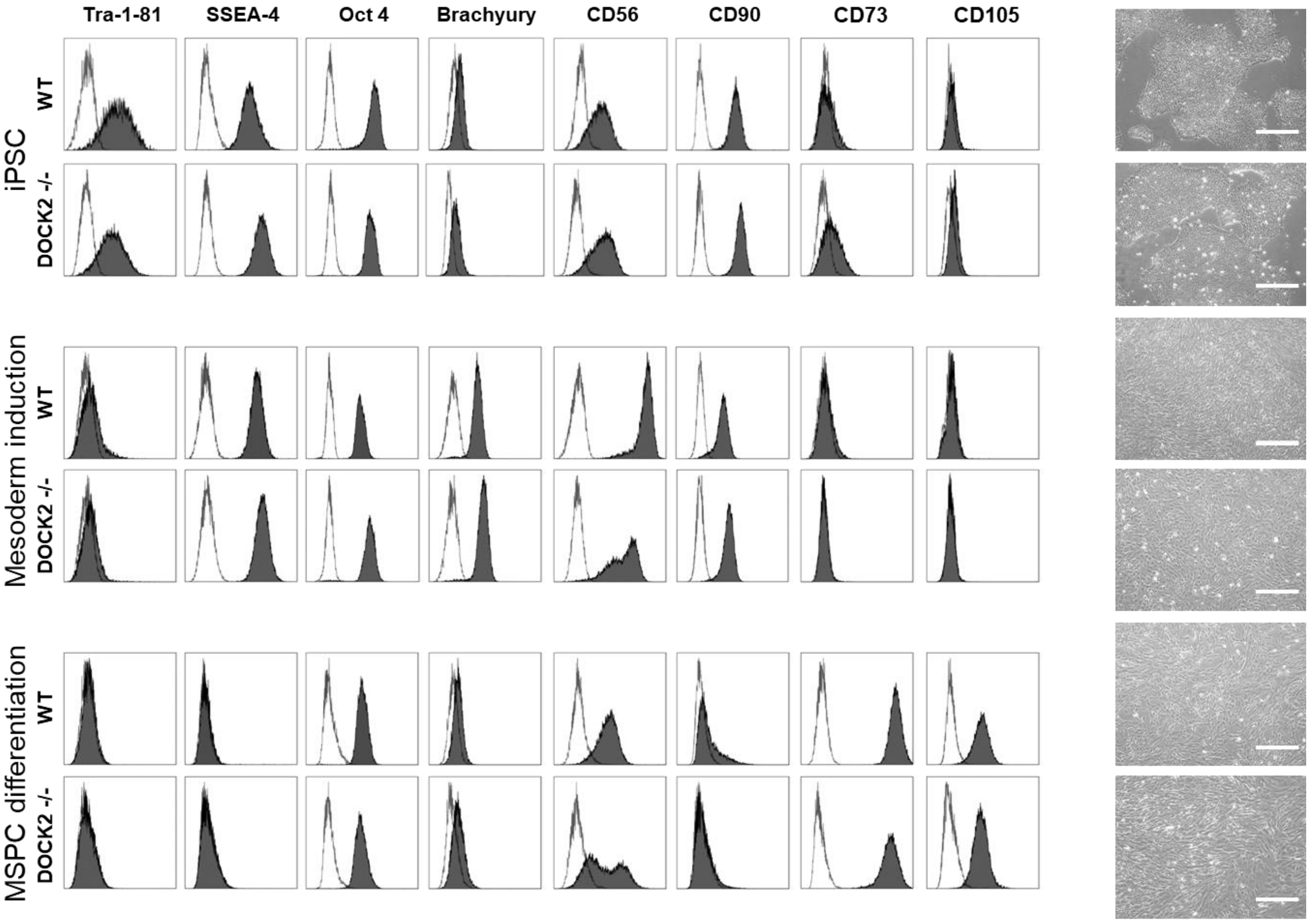

Supplementary Fig. 9b: Maturation of iPS-MSPCs. Representative flow cytometry profiles over time of differentiation; depicted as described in Fig.9a. Representative phase-contrast microscopy pictures (right; scale bar 400 µm). An additional maturation beyond passage 8 in MSPC-optimized medium is monitored[29].

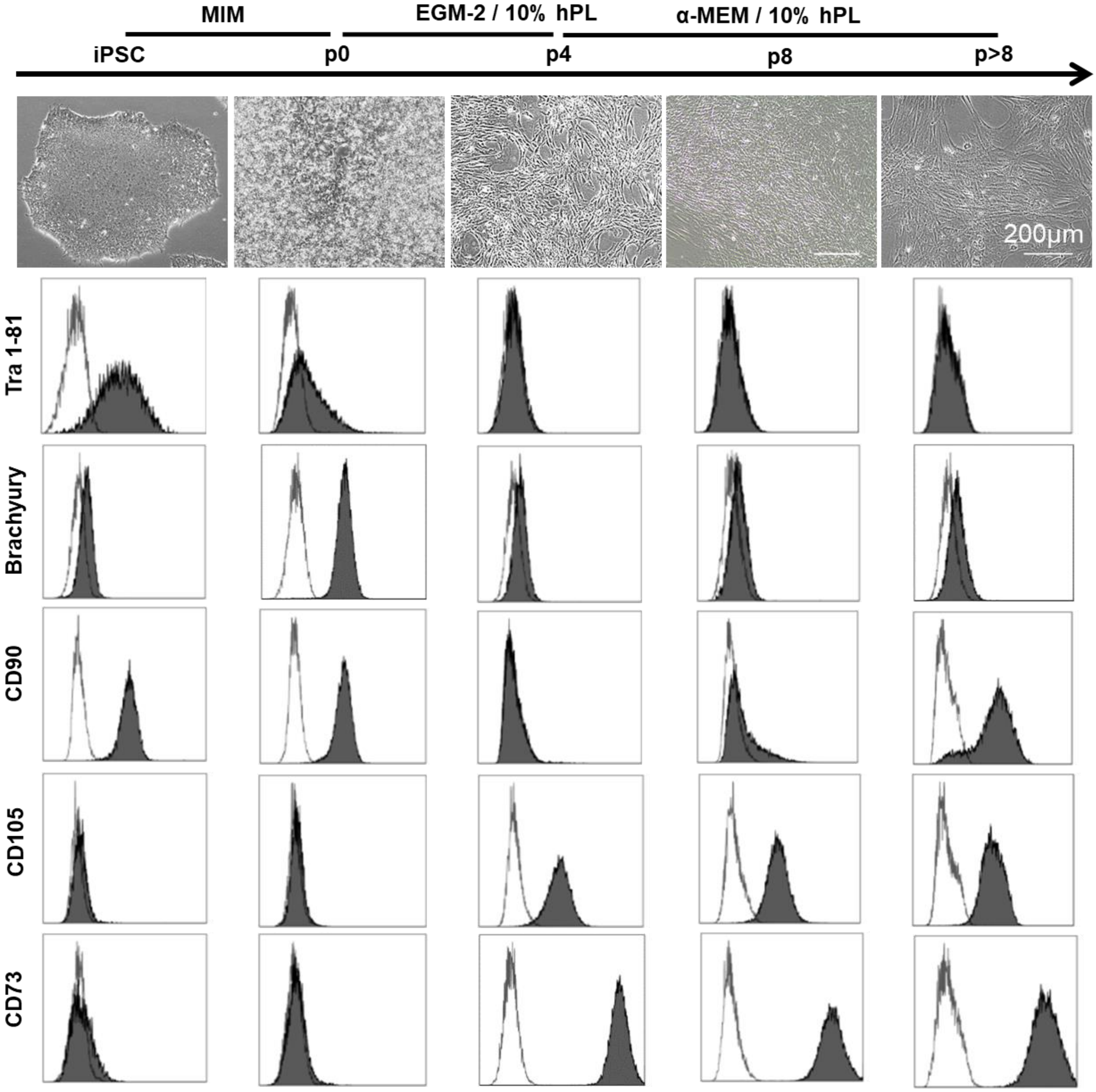

Supplementary Fig. 9c: Marker expression profile of *DOCK2* knockout iPSC (CRISPR/Cas iPS-MSPC *DOCK2*$^{-/-}$), their mesoderm induction and differentiation, compared to wild type (WT) controls. Representative histograms of flow cytometric analysis of WT iPSCs (reprogrammed from UCB-derived MSPCs) and *DOCK2*$^{-/-}$ iPS-MSPCs (CRISPR/Cas-derived, one out of three clones is shown), after mesoderm induction and further differentiated iPS-MSPC progeny are depicted. Histograms show fluorescent cell surface (anti-Tra 1-81, SSEA-4, CD56, CD90, CD73 and CD105) and intracellular (anti-brachyury and anti-Oct4) staining intensity of monoclonal antibodies conjugated to fluorophores (gray shading) and their corresponding isotype control (no shading). Histograms show the populations obtained following the hierarchical gating strategy: size and granularity, doublet exclusion, live cell population and corresponding marker. Increased brachyury and CD56 expression compared to iPSC indicated mesoderm induction, *DOCK2*$^{-/-}$ iPS-MSPCs did not show a uniform CD56 expressing population. Mesoderm differentiated *DOCK2*$^{-/-}$ iPS-MSPCs lost Tra-1-81and SSEA-4 expression and induced CD73 and CD105 comparable to WT iPS-MSPCs. The morphology of WT and *DOCK2*$^{-/-}$ iPS-MSPCs was comparable (representative pictures; scale bar = 400 µm).

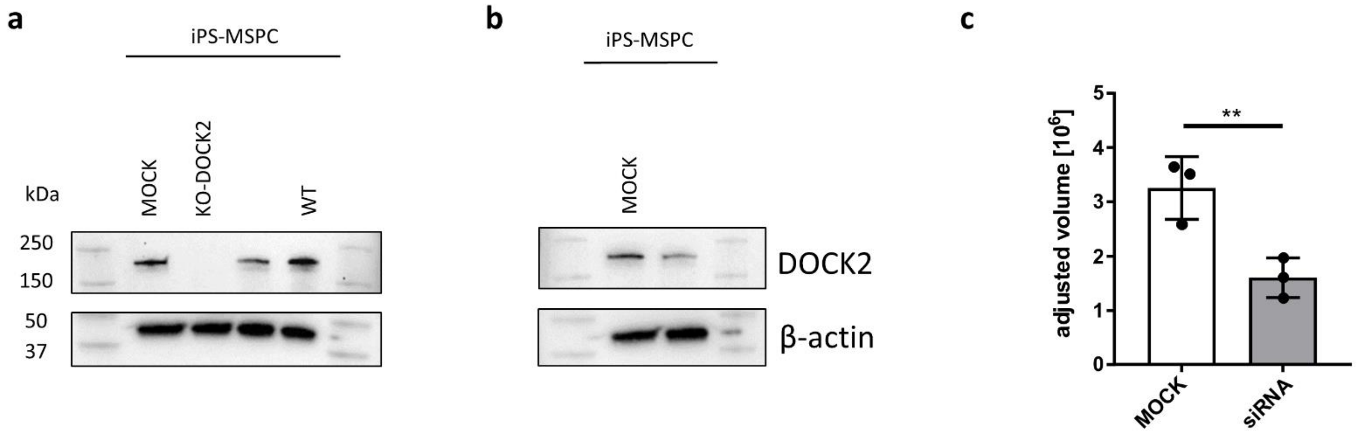

Supplementary Fig. 10: Reduced expression of DOCK2 after DOCK2 siRNA transfection of mesoderm induced iPS cells. (a) Comparison of DOCK2 expression levels between MOCK-transfected, *DOCK2* knockout, DOCK2 siRNA-transfected and wild type (WT) MSPC. (b) Transfection of iPS-MSPC with DOCK2 siRNA 72h prior to immunomodulation assays showed a reduced expression of DOCK2 compared to MOCK transfection. (c) Normalization of signal density in (b) using b-actin as loading control showing a 2.03-fold reduction from 3.25 to 1.06 (adjusted volume 10^6). Paired t test ** $p< 0.01$ (n=3). Error bars represent standard deviation.

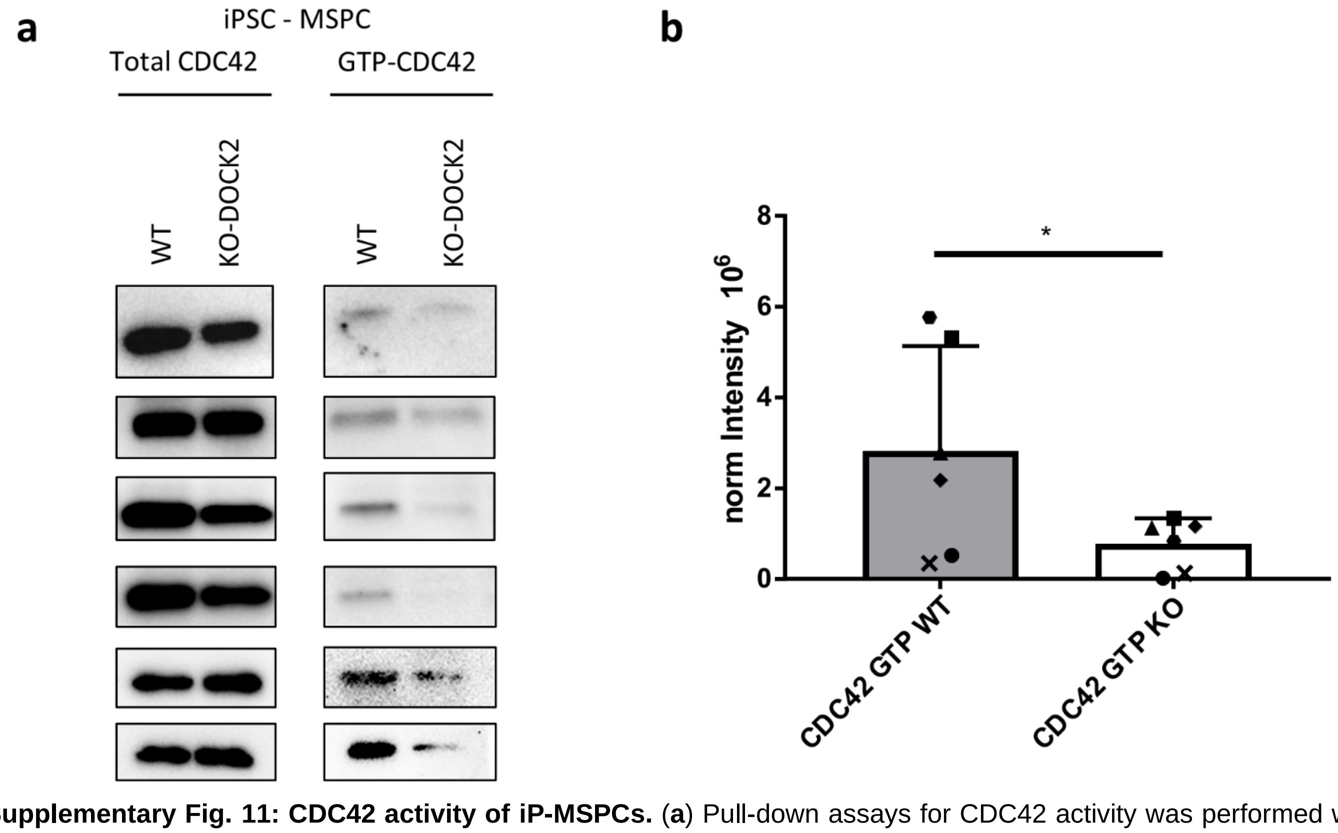

Supplementary Fig. 11: CDC42 activity of iP-MSPCs. (**a**) Pull-down assays for CDC42 activity was performed with cell lysates of wild type (WT) compared to *DOCK2* knockout (KD) iPS-MSPCs. (b) Quantitation of Western blots is shown, and the ratio of total CDC42 and GTP-CDC42 was calculated. Paired t test * $p< 0.05$ (n = 6). Error bars represent standard deviation.

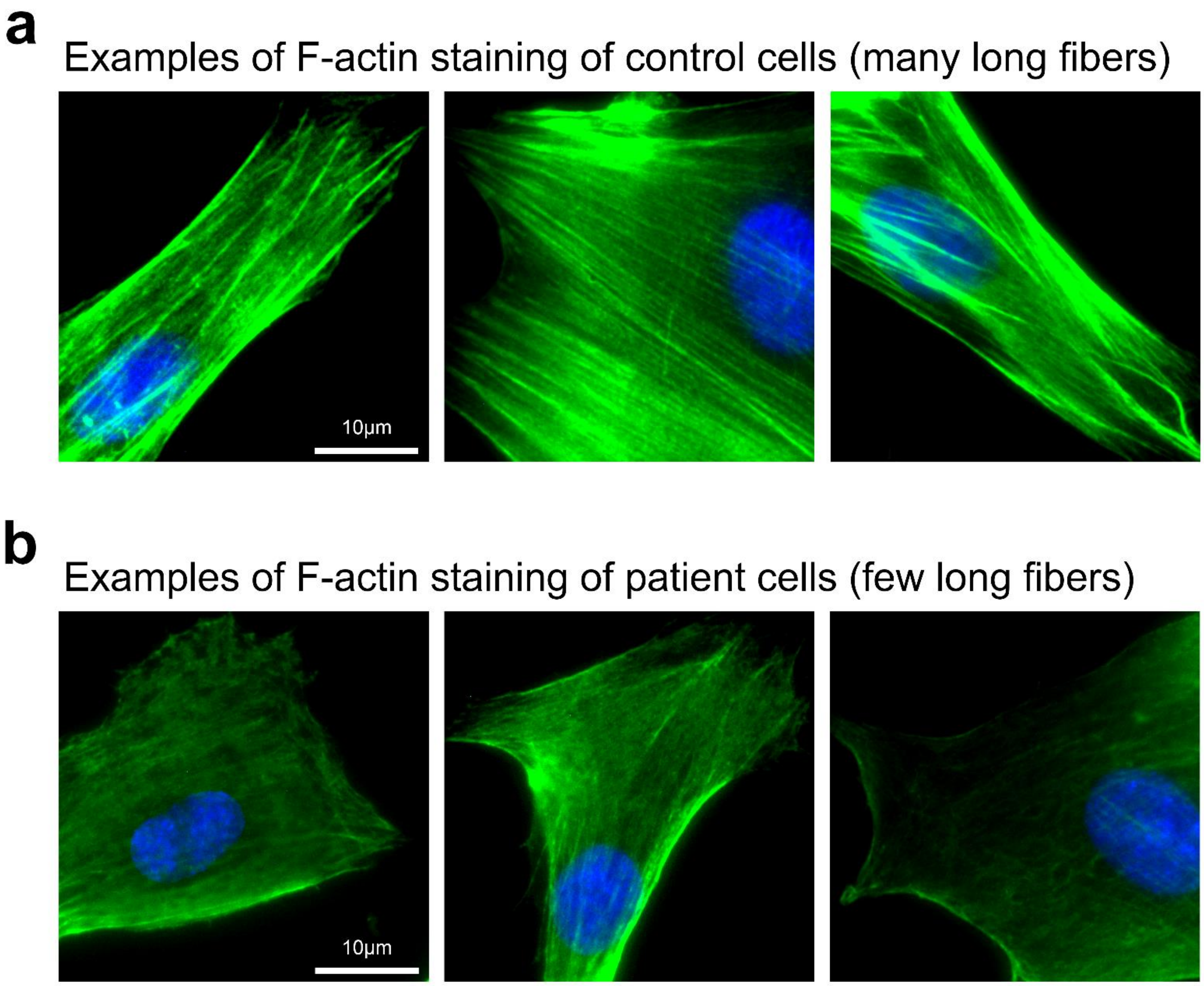

Supplementary Fig. 12: Representative F-actin staining. (a) Skin fibroblasts from healthy controls (n = 2) and (b) *DOCK2* mutant patients (n = 2) were stained with phalloidin and the presence of long stress fibers was enumerated (see Fig.4a).

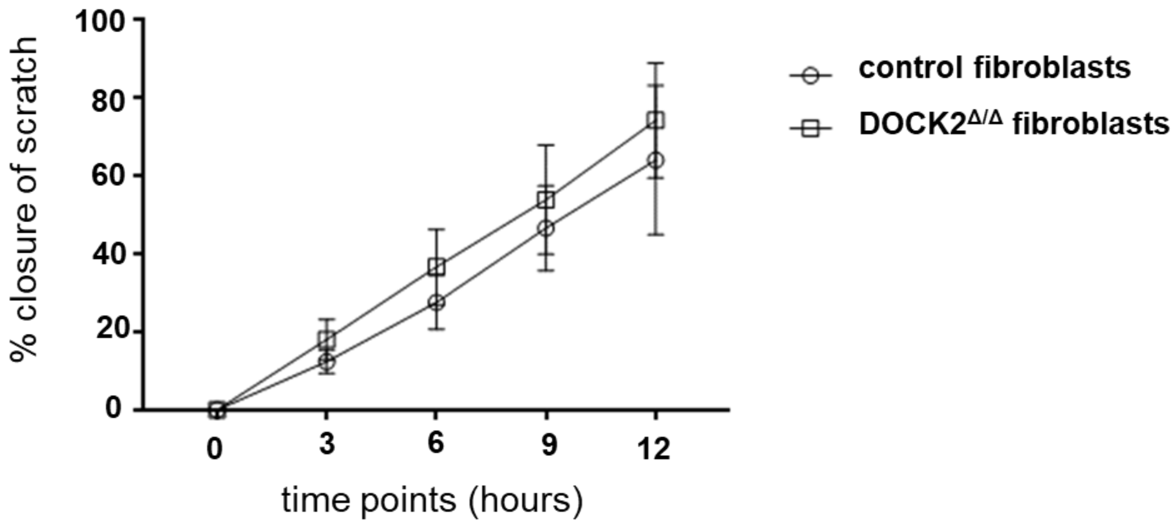

Supplementary Fig. 13: **Scratch assay of fibroblasts derived from DOCK2 deficient patients vs. controls.** Wounding a fibroblast monolayer in a scratch assay was used to test migratory wound repair of DOCK2 deficient fibroblasts compared to control fibroblasts. The assay was repeated three times with two different patients and two different control cells. DOCK2 deficient patient cells did not show an altered wound closure capacity as analyzed by 2-way ANOVA with Sidak correction for multiple comparisons. n = 6, F = 2.067, DF = 1, p(3h) = 0.8825, p(6h) = 0.5207, p(9h) = 0.7225, p(12h) = 0.3952. Error bars represent standard deviation.

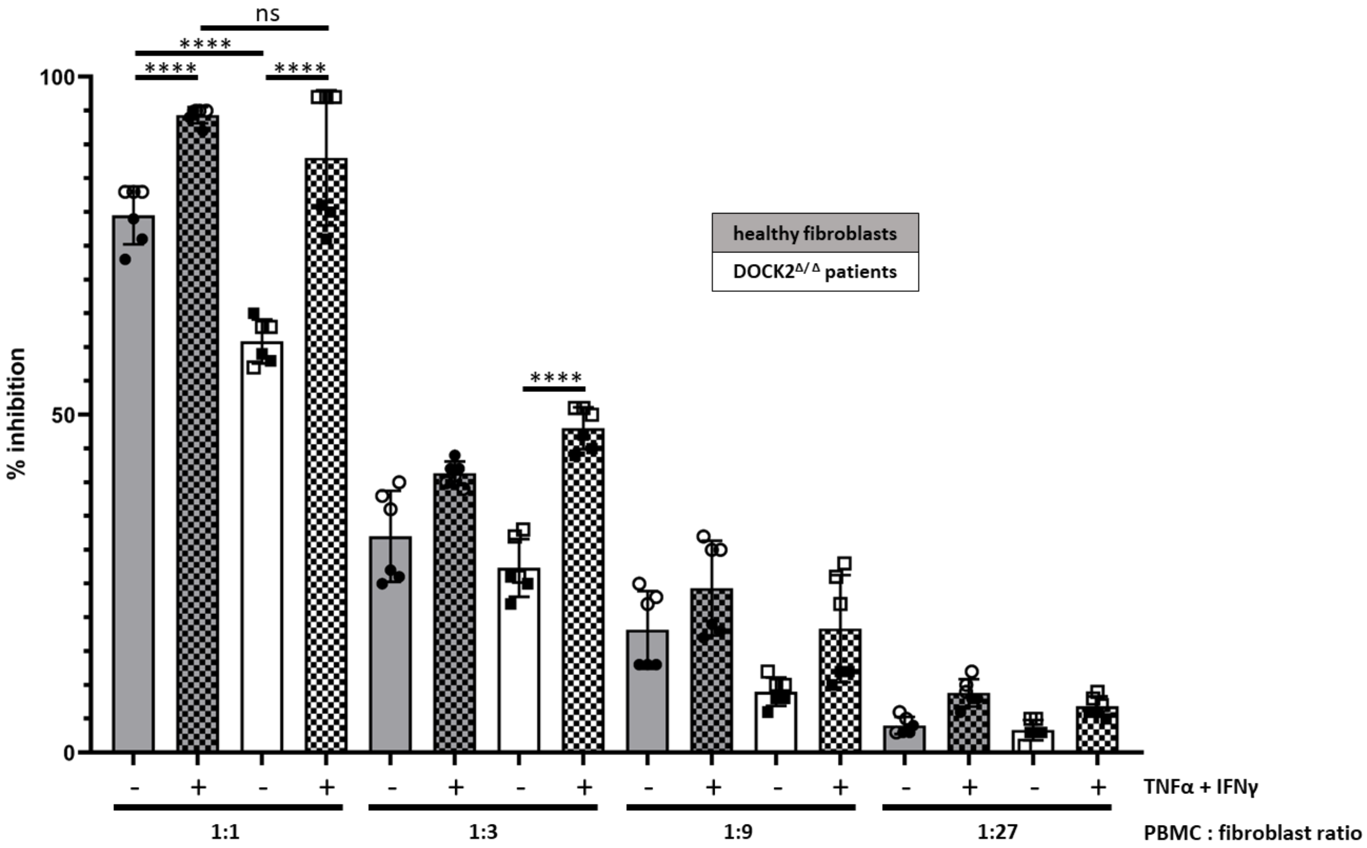

Supplementary Fig. 14: Pro-inflammatory signaling ameliorates deficient immunomodulation. Healthy control fibroblasts and *DOCK2*$^{\Delta/\Delta}$ patient-derived fibroblast lines were compared for their immunomodulatory capacity inhibiting PHA-induced mitogenesis of human CD3^{+} T cells among PBMCs, in the absence or presence of titrated amounts of human recombinant TNF-α and IFN-γ, in a dose-dependent manner. Pooled data from two healthy donor and two DOCK2$^{\Delta/\Delta}$ patient fibroblasts in independent assays (n = 6), one way ANOVA with multiple comparisons. Error bars represent standard deviation.

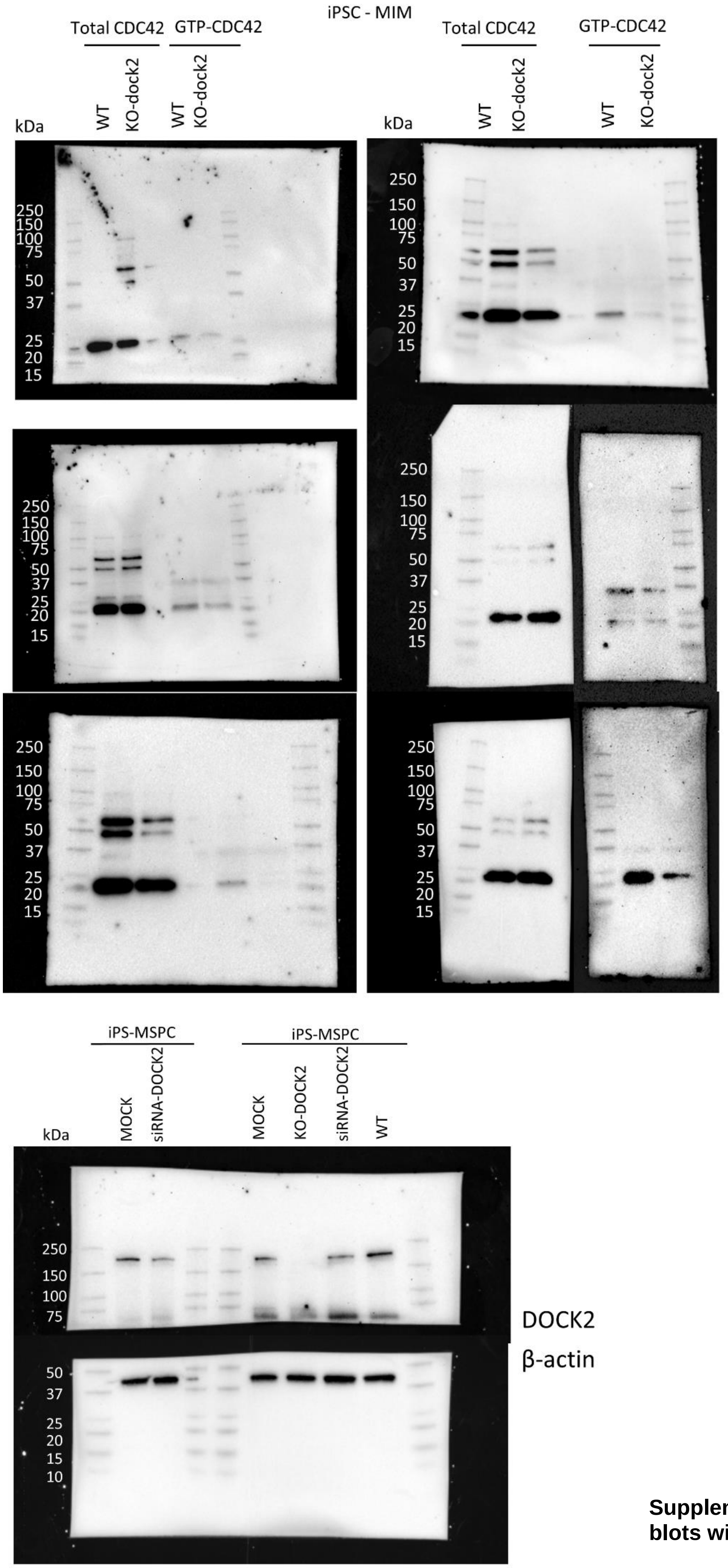

Supplementary Fig. 15: Uncropped Western blots with size markers.

Supplementary Table 1

Reference	Small molecule Differentiation Induction	Proliferation	CFU-F	Immune phenotype		3-lin differentiation		Function		Model Regeneration Immune modulation	3D	Scalable production	Compared to primary cells	RNAseq/Methylome analysis
				CD73/90 /105	+	in vitro	in vivo	in vitro	in vivo					
1	SB431542	✓	-	✓	✓	✓	-	-	-	-	-	-		
2	-	-	-	✓	-	-	-	✓	✓	effect on islet allograft survival +/- Rapamycin, Treg induction in mice	-	-	-	-
3	-	✓	-	✓	-	✓	-	-	✓	osteochondral repair	-	-	-	-
4	-	✓	-	✓	-	✓	-	-	-	direct comparison to parental BM-MSPC	EB	-	-	-
5	-	✓	✓	✓	✓	✓	-	-	-	T Cell inhibition but iMSC less potent than primary MSC, DNAm profile	EB	-	✓	Affymetrix array, Infinium HumanMethylation450 BeadChip
6	-	-	-	-	-	-	-	✓	-	T cell inhibition (PHA) similar to primary MSPC, Treg induction and cytokine production in Derp1 stimulated PBMCs (allergy), function associated with PGE2 production	-	-	✓	-
7	CHIR99021, SB431542	-	-	no CD90	-	✓	-	-	-	-	-	-	-	-
8	-	-	-	✓	✓	✓	-	✓	-	iPS-MSPC impair NK cell cytotoxicity, proliferation and activation	-	-	✓	-
9	-	-	-	✓	✓	✓	-	-	-	-	-	-	-	-
10	-	-	-	✓	✓	✓	✓	-	-	-	-	-	-	-
11	-	-	-	✓	✓	only osteogenic	-	-	-	-	-	-	-	-
12	-	✓	-	✓	✓	✓	-	-	-	-	-	-	-	-
13	-	-	-	-	-	✓	-	-	-	-	EB	-	-	-
14	-	-	-	✓	✓	✓	-	✓	✓	hind limb ischemia	-	-	✓	-
15	-	-	-	-	-	-	-	-	✓	myocardium infarct	EB	-	-	--
16	-	-	-	✓	-	✓	-	-	✓	hepatic failure	-	-	✓	
17	-	✓	-	✓	-	✓	-	✓	-	CD34+ support, CD4+ T cell inhibition MLR, cytokine secretion, increased CD25+	-	-	✓	-
18	-	-	-	✓	-	✓	-	-	✓	osteochondral defects	EB	-	-	-
19	CHIR99021	-	-	no CD90	✓	-	-	✓	-	network EC-pericyte	-	-	-	-
20	CP21R7, BMP4, PDGF-BB, activin A	-	-	-	✓	-	-	✓	✓	vessel formation of vSM	-	-	-	transcriptomics, metabolomics – signature similar to primary vascular cells
21	-	✓	✓	no CD73	-	✓	✓	-	-	osteogenesis	EB	-	✓	iPS reprogrammed from dermal fibroblasts, RNAseq profile of BM-MSPC, iPS-MSPC vs. reference profiles
22	-	-	-	no CD73, no CD105	-	✓	-	-	✓	inflammatory bowel disease model	-	-	✓	-
23	SB-431542	-	-	✓	-	✓	-	-	-	-	EB	-	✓	human gene expression chip microarray – ageing signature and rejuvenation
24	-	-	-	-	-	-	-	-	✓	airway inflammation	-	-	✓	-
25	-	-	-	no CD90	-	✓	-	✓	✓	bone defect model	EB	-	✓	-
26	SB203580	✓	-	✓	✓	✓	-	✓	-	wound healing (scratch assay), angiogenesis (matrigel tube formation assay)	EB	-	✓	-
27	SB431542	✓	✓	✓	✓	✓	-	✓	✓	tumour homing	-	-	-	-
28	-	-	-	✓	-	✓	-	✓	✓	osteogenesis	-	-	-	-
29	-	-	-	✓	✓	✓	-	✓	✓	inhibition of T cell proliferation as by BM-derived stromal cells therapeutic efficacy in murine sepsis model	-	-	✓	analysis of c-Myc and downstream pathway by cDNA microarray
30	-	-	-	✓	✓	-	-	-	✓	phase 1 open-label clinical trial in subjects with steroid-resistant acute graft versus host disease comparing dose-escalation	-	✓	-	mRNAseq global gene expression (transcriptome) analysis

Supplementary Table 2: Genes linked with T-cell proliferation (**red**) or G-protein signalling (**blue**) found significantly upregulated in iPS-MSPC p8 compared to p0. P values were adjusted using Benjamini-Hochberg correction.

Gene name	Log2 FC p8 vs p0	P. adj	Gene description	Function
DOCK10	7,36	1,25E-153	Dedicator of cytokinesis 10 (Zizimin3)	CDC42-spec. small G protein-activating DOCK-D GEF (hemato.-restricted)
C3AR1	6,68	3,23E-75	Complement C3a receptor 1	Anaphylatoxin binding in immunity & cancer
PPARG	5,25	4,69E-234	Peroxisome proliferator activated receptor γ	Type II nuclear rec. (glitazone); fatty acid & glucose metabolism
PLD1	4,84	2,29E-144	Phospholipase D1	Phyphatidylcholine hydrolysis; interacting with CDC42, RhoA; α-Syn.
RAB7B	4,09	3,41E-20	RAB7B, RAS oncogene family member	
GPR68	3,79	1,08E-58	G protein-coupled receptor 68	
ADGRE3	3,02	1,29E-205	adhesion G protein-coupled receptor E3	
RAP1B	2,45	3,77E-112	RAP1B, member of RAS oncogene family	
PAK3	2,30	8,32E-27	p21 (RAC1) activated kinase 3	
CD44	2,24	2,63E-26	Cell surface glycoprotein (Indian blood group)	Hyaluronan rec. (Pgp1; HCAM, HCELL); lymphocyte migration/homing
VCAM1	11,82	1,24E-47	CD106, vascular cell adhesion molecule 1	VLA-4 ($\alpha 4 \beta 1$ integrin, CD49d/CD29) ligand sialoglycoprotein; leukocyte-EC binding (NFκB)
IL6	7,30	1,30E-35	interleukin 6	
CDKN2A	7,10	5,53E-171	cyclin dependent kinase inhibitor 2A	
TNFSF18	6,62	2,28E-22	TNF superfamily member 18	
TNFSF4	6,46	1,24E-90	TNF superfamily member 4	
RIPK3	6,35	7,16E-27	receptor interacting serine/threonine kinase 3	
DOCK2	6,01	6,80E-247	Dedicator of cytokinesis 2	Rac1-spec. Rho-activating DOCK-A GEF (hemato. Restricted)
ANXA1	5,88	8,22E-112	Annexin A1	
PDCD1LG2	5,50	5,92E-106	**CD273**, programmed cell death 1 ligand 2	PD-L2
TNFRSF14	5,14	7,05E-23	TNF receptor superfamily member 14	
TNFSF8	4,93	2,61E-07	TNF superfamily member 8	
CD274	4,74	3,04E-49	PD-L1 molecule	
TGFBR2	4,51	3,11E-136	transforming growth factor beta receptor 2	
SDC4	4,34	0,00E+00	syndecan 4	
LGALS3	4,33	1,15E-118	galectin 3	
IL12A	4,20	6,44E-44	interleukin 12A	
ELF4	4,10	1,32E-140	E74 like ETS transcription factor 4	
PYCARD	3,52	9,63E-79	PYD and CARD domain containing	
PRNP	3,40	2,86E-128	prion protein	
RAC2	3,34	4,60E-60	Rac family small GTPase 2	
IRF1	3,17	1,25E-131	interferon regulatory factor 1	
IL6ST	3,13	1,49E-88	interleukin 6 signal transducer	
CARD11	2,99	1,55E-16	caspase recruitment domain family member 11	
CCND3	2,98	3,15E-113	cyclin D3	
CD55	2,85	8,48E-35	CD55 molecule (Cromer blood group)	
LRRC32	2,82	4,27E-29	leucine rich repeat containing 32	
BTN3A1	2,70	3,31E-44	butyrophilin subfamily 3 member A1	
PSMB10	2,59	3,87E-72	proteasome subunit beta 10	
SCGB1A1	2,33	2,66E-07	secretoglobin family 1A member 1	
SH3RF1	2,26	4,14E-89	SH3 domain containing ring finger 1	
HLA-G	2,21	1,25E-19	major histocompatibility complex, class I, G	
IL15	2,16	1,35E-38	interleukin 15	
CD151	2,15	5,00E-47	CD151 molecule (Raph blood group)	
GPNMB	2,10	4,28E-11	glycoprotein nmb	
CEBPB	2,06	3,20E-09	CCAAT/enhancer binding protein beta	
VSIR	1,81	7,11E-09	V-set immunoregulatory receptor	
RIPK2	1,62	4,97E-35	receptor interacting serine/threonine kinase 2	
TGFB1	1,58	6,53E-103	transforming growth factor beta 1	
HLA-E	1,44	2,88E-30	major histocompatibility complex, class I, E	
FADD	1,25	1,19E-26	Fas associated via death domain	
NCSTN	1,20	2,45E-64	nicastrin	
PRKAR1A	1,16	4,98E-34	protein kinase cAMP-dep. type I regul. subunit α	

Supplementary Table 3

**Guide sequences**	
guide-1 (DOCK2-Ex37-1-Corr)	crRNA: CACAGTCCAGGTGAAGATCG
guide-2 (DOCK2-Ex37-2-Corr)	crRNA: GAAGATCGCGGAGTTTGTAC
**PCR primers for amplification of target site**	
DOCK Ex37-86 left	GGACTTTCTTGCGACCCAGA
DOCK Ex37-86 right	ATGTGACCAGGGAAGCAGTG

Supplementary References:

1. Chen, Y. S. *et al.* Small molecule mesengenic induction of human induced pluripotent stem cells to generate mesenchymal stem/stromal cells. *Stem Cells Transl. Med.* **1**, 83–95 (2012).
2. Cheng, P.-P. *et al.* iPSC-MSCs Combined with Low-Dose Rapamycin Induced Islet Allograft Tolerance Through Suppressing Th1 and Enhancing Regulatory T-Cell Differentiation. *Stem Cells Dev.* **24**, 1793–1804 (2015).
3. Chijimatsu, R. *et al.* Characterization of Mesenchymal Stem Cell-Like Cells Derived From Human iPSCs via Neural Crest Development and Their Application for Osteochondral Repair. *Stem Cells Int.* **2017**, 1960965 (2017).
4. Diederichs, S. & Tuan, R. S. Functional comparison of human-induced pluripotent stem cell-derived mesenchymal cells and bone marrow-derived mesenchymal stromal cells from the same donor. *Stem Cells Dev.* **23**, 1594–1610 (2014).
5. Frobel, J. *et al.* Epigenetic rejuvenation of mesenchymal stromal cells derived from induced pluripotent stem cells. *Stem cell reports* **3**, 414–422 (2014).
6. Fu, Q. L. *et al.* Mesenchymal stem cells derived from human induced pluripotent stem cells modulate T-cell phenotypes in allergic rhinitis. *Allergy* **67**, 1215–1222 (2012).
7. Fukuta, M. *et al.* Derivation of mesenchymal stromal cells from pluripotent stem cells through a neural crest lineage using small molecule compounds with defined media. *PLoS One* **9**, e112291–e112291 (2014).
8. Giuliani, M. *et al.* Human mesenchymal stem cells derived from induced pluripotent stem cells down-regulate NK-cell cytolytic machinery. *Blood* **118**, 3254–3262 (2011).
9. Guzzo, R. M., Gibson, J., Xu, R.-H., Lee, F. Y. & Drissi, H. Efficient differentiation of human iPSC-derived mesenchymal stem cells to chondroprogenitor cells. *J. Cell. Biochem.* **114**, 480–490 (2013).
10. Hynes, K., Menicanin, D., Mrozik, K., Gronthos, S. & Bartold, P. M. Generation of functional mesenchymal stem cells from different induced pluripotent stem cell lines. *Stem Cells Dev.* **23**, 1084–1096 (2014).
11. Ishiy, F. A. A. *et al.* Improvement of *In Vitro* Osteogenic Potential through Differentiation of Induced Pluripotent Stem Cells from Human Exfoliated Dental Tissue towards Mesenchymal-Like Stem Cells. *Stem Cells Int.* **2015**, 249098 (2015).
12. Kang, R. *et al.* Mesenchymal stem cells derived from human induced pluripotent stem cells retain adequate osteogenicity and chondrogenicity but less adipogenicity. *Stem Cell Res. Ther.* **6**, 144 (2015).
13. Lee, T.-J. *et al.* Mesenchymal stem cell-conditioned medium enhances osteogenic and chondrogenic differentiation of human embryonic stem cells and human induced pluripotent stem cells by mesodermal lineage induction. *Tissue Eng. Part A* **20**, 1306–1313 (2014).
14. Lian, Q. *et al.* Functional Mesenchymal Stem Cells Derived From Human Induced Pluripotent Stem Cells Attenuate Limb Ischemia in Mice. *Circulation* **121**, 1113–1123 (2010).
15. Miao, Q. *et al.* iPSC-derived human mesenchymal stem cells improve myocardial strain of infarcted myocardium. *J. Cell. Mol. Med.* **18**, 1644–1654 (2014).
16. Moslem, M., Eberle, I., Weber, I., Henschler, R. & Cantz, T. Mesenchymal Stem/Stromal Cells Derived from Induced Pluripotent Stem Cells Support CD34(pos) Hematopoietic Stem Cell Propagation and Suppress Inflammatory Reaction. *Stem Cells Int.* **2015**, 843058 (2015).
17. Moslem, M., Valojerdi, M. R., Pournasr, B., Muhammadnejad, A. & Baharvand, H. Therapeutic Potential of Human Induced Pluripotent Stem Cell-Derived Mesenchymal Stem Cells in Mice with Lethal Fulminant Hepatic Failure. *Cell Transplant.* **22**, 1785–1799 (2013).
18. Nejadnik, H. *et al.* Improved approach for chondrogenic differentiation of human induced pluripotent stem cells. *Stem cell Rev. reports* **11**, 242–253 (2015).
19. Orlova, V. V *et al.* Functionality of Endothelial Cells and Pericytes From Human Pluripotent Stem Cells Demonstrated in Cultured Vascular Plexus and Zebrafish Xenografts. *Arterioscler. Thromb. Vasc. Biol.* **34**, 177–186 (2014).
20. Patsch, C. *et al.* Generation of vascular endothelial and smooth muscle cells from human pluripotent stem cells. *Nat. Cell Biol.* **17**, 994–1003 (2015).

21. Sheyn, D. *et al.* Human Induced Pluripotent Stem Cells Differentiate Into Functional Mesenchymal Stem Cells and Repair Bone Defects. *Stem Cells Transl. Med.* **5**, 1447–1460 (2016).
22. Soontararak, S. *et al.* Mesenchymal Stem Cells (MSC) Derived from Induced Pluripotent Stem Cells (iPSC) Equivalent to Adipose-Derived MSC in Promoting Intestinal Healing and Microbiome Normalization in Mouse Inflammatory Bowel Disease Model. *Stem Cells Transl. Med.* **7**, 456–467 (2018).
23. Spitzhorn, L.-S. *et al.* Human iPSC-derived MSCs (iMSCs) from aged individuals acquire a rejuvenation signature. *Stem Cell Res. Ther.* **10**, 100 (2019).
24. Sun, Y.-Q. *et al.* Human pluripotent stem cell-derived mesenchymal stem cells prevent allergic airway inflammation in mice. *Stem Cells* **30**, 2692–2699 (2012).
25. Villa-Diaz, L. G. *et al.* Derivation of mesenchymal stem cells from human induced pluripotent stem cells cultured on synthetic substrates. *Stem Cells* **30**, 1174–1181 (2012).
26. Wei, H. *et al.* One-step derivation of cardiomyocytes and mesenchymal stem cells from human pluripotent stem cells. *Stem Cell Res.* **9**, 87–100 (2012).
27. Zhao, Q. *et al.* MSCs derived from iPSCs with a modified protocol are tumor-tropic but have much less potential to promote tumors than bone marrow MSCs. *Proc. Natl. Acad. Sci. U. S. A.* **112**, 530–535 (2015).
28. Zou, L. *et al.* A simple method for deriving functional MSCs and applied for osteogenesis in 3D scaffolds. *Sci. Rep.* **3**, 2243 (2013).
29. Wang L.-T. *et al*. Differentiation of Mesenchymal Stem Cells from Human Induced Pluripotent Stem Cells Results in Downregulation of c-Myc and DNA Replication Pathways with Immunomodulation Toward CD4 and CD8 Cells. Stem Cells **36**, 903 (2018).
30. Bloor A.J.C. *et al*. Production, safety and efficacy of iPSC-derived mesenchymal stromal cells in acute steroid-resistant graft versus host disease: a phase I, multicenter, open-label, dose-escalation study. Nat Med 26, 1720 (2020).
