# Supplementary material for: Extra-hematopoietic immunomodulatory role of the guanine-exchange factor DOCK2
Source: Commun Biol. 2022 Nov 15;5:1246. doi: 10.1038/s42003-022-04078-1 (PMC9666545; doi:10.1038/s42003-022-04078-1)
Supplement: Supplementary file 5 — Reporting Summary [file 42003_2022_4078_MOESM5_ESM.pdf]

nature portfolio

Corresponding author(s): Prof. Dirk Strunk

Last updated by author(s): Nov 24, 2021

# Reporting Summary

Nature Portfolio wishes to improve the reproducibility of the work that we publish. This form provides structure for consistency and transparency in reporting. For further information on Nature Portfolio policies, see our Editorial Policies and the Editorial Policy Checklist.

## Statistics

For all statistical analyses, confirm that the following items are present in the figure legend, table legend, main text, or Methods section.

| n/a | Confirmed | |
|---|---|---|
| ☐ | ☒ | The exact sample size (*n*) for each experimental group/condition, given as a discrete number and unit of measurement |
| ☐ | ☒ | A statement on whether measurements were taken from distinct samples or whether the same sample was measured repeatedly |
| ☐ | ☒ | The statistical test(s) used AND whether they are one- or two-sided<br>*Only common tests should be described solely by name; describe more complex techniques in the Methods section.* |
| ☒ | ☐ | A description of all covariates tested |
| ☒ | ☐ | A description of any assumptions or corrections, such as tests of normality and adjustment for multiple comparisons |
| ☐ | ☒ | A full description of the statistical parameters including central tendency (e.g. means) or other basic estimates (e.g. regression coefficient) AND variation (e.g. standard deviation) or associated estimates of uncertainty (e.g. confidence intervals) |
| ☐ | ☒ | For null hypothesis testing, the test statistic (e.g. *F*, *t*, *r*) with confidence intervals, effect sizes, degrees of freedom and *P* value noted<br>*Give P values as exact values whenever suitable.* |
| ☒ | ☐ | For Bayesian analysis, information on the choice of priors and Markov chain Monte Carlo settings |
| ☒ | ☐ | For hierarchical and complex designs, identification of the appropriate level for tests and full reporting of outcomes |
| ☒ | ☐ | Estimates of effect sizes (e.g. Cohen's *d*, Pearson's *r*), indicating how they were calculated |

*Our web collection on statistics for biologists contains articles on many of the points above.*

## Software and code

Policy information about availability of computer code

| | |
|---|---|
| Data collection | Five-laser BD LSR-FortessaTM (BD Bioscience) with BD FACSDiva Software 8.0.1 Firmware version 1.4 were used to aquire flow cytometry data. T cell proliferation was analyzed using Gallios 10-color flow cytometer and the Kaluza G1.0 software (both Coulter)<br>Eclipse Ti inverted microscope (Nikon) with a customized live cell incubation system (Oko lab) and NIS elements imaging software AR 4.30.02 (Nikon), laser scanning microscopes Axio Observer Z1 attached to LSM710 (Carl Zeiss) and ZEN 2012 software (blue edition, Carl Zeiss), EVOS XL microscope (Thermo Fisher), VS-120-L Olympus slide scanner 100-W system and Olympus VS-ASW-L100 program were used to aquire images. |
| Data analysis | Kaluza analysis software version 1.3.14026.13330 (Beckman Coulter) was used to analyse flow cytometry data.<br>Statistics was calculated in GraphPadPrism7, R (3.6.3).<br>RNAseq and MethylCap seq data was analyzed using R software (3.6.3)<br>Figures were prepared using Microsoft Power Point 2016 and Inkscape. |

For manuscripts utilizing custom algorithms or software that are central to the research but not yet described in published literature, software must be made available to editors and reviewers. We strongly encourage code deposition in a community repository (e.g. GitHub). See the Nature Portfolio guidelines for submitting code & software for further information.



March 2021

## Data

Policy information about availability of data

All manuscripts must include a data availability statement. This statement should provide the following information, where applicable:

- Accession codes, unique identifiers, or web links for publicly available datasets
- A description of any restrictions on data availability
- For clinical datasets or third party data, please ensure that the statement adheres to our policy

All data supporting the findings of the present study are available from the corresponding author on reasonable request. RNAseq and MethylCap-seq raw data are available on GEO database (GEO ID provided in the manuscript)

# Field-specific reporting

Please select the one below that is the best fit for your research. If you are not sure, read the appropriate sections before making your selection.

☒ Life sciences ☐ Behavioural & social sciences ☐ Ecological, evolutionary & environmental sciences

For a reference copy of the document with all sections, see nature.com/documents/nr-reporting-summary-flat.pdf

# Life sciences study design

All studies must disclose on these points even when the disclosure is negative.

| | |
|---|---|
| Sample size | For experiments involving characterization of adult cells n=3 was chosen as minimal replicate number, including different cell donors. Human pluripotent stem cell experiments were performed at various timepoints of at least two independent donors. |
| Data exclusions | no data excluded |
| Replication | Experiments were successfully replicated at different time points using different cell donors, different hPL batches |
| Randomization | All samples were analyzed equally with no sub-sampling and thus, there was no requirement for randomization |
| Blinding | there was no requirement for blinding |

# Reporting for specific materials, systems and methods

We require information from authors about some types of materials, experimental systems and methods used in many studies. Here, indicate whether each material, system or method listed is relevant to your study. If you are not sure if a list item applies to your research, read the appropriate section before selecting a response.

### Materials & experimental systems

| n/a | Involved in the study | |
|---|---|---|
| ☐ | ☒ | Antibodies |
| ☐ | ☒ | Eukaryotic cell lines |
| ☒ | ☐ | Palaeontology and archaeology |
| ☐ | ☒ | Animals and other organisms |
| ☒ | ☐ | Human research participants |
| ☒ | ☐ | Clinical data |
| ☒ | ☐ | Dual use research of concern |

### Methods

| n/a | Involved in the study | |
|---|---|---|
| ☒ | ☐ | ChIP-seq |
| ☐ | ☒ | Flow cytometry |
| ☒ | ☐ | MRI-based neuroimaging |

## Antibodies

Antibodies used

(1) Anti-Tra 1-81-AF647, mouse monoclonal; clone: TRA-1-81; BD Biosciences; 560793; lot # 6036678
2) Anti-SSEA-4-PE, mouse monoclonal; clone: MC813-70; BD Biosciences; 560128; lot # 77229
3) Anti-OCT4-PE, mouse monoclonal; clone: 3A2A20; biolegend; 653704; lot #.: 653704
4) Anti-OCT4-FITC
5) Anti-CD56-PE, mouse monoclonal; clone CMSSB; eBioscience; 12-0567; lot # 43041328
6) Anti-CD90-BUV395, mouse monoclonal; clone 5E10; BD Biosciences; 563804; lot # 4290881
7) Anti-CD105-eFluor450, mouse monoclonal; clone SN6; eBioscience; 48-1057; lot # E16329-102
8) Anti-CD073-PE; mouse monoclonal; clone AD2; BD Biosciences; 550257; lot # 3319691
9) Anti-CD146-PE-Vio 770; mouse monoclonal; clone 541-10B2; Miltenyi; 130-099-956; lot # 5141001212
13) Anti-CD045-APC; mouse monoclonal; clone HI30; BD Biosciences; 555485: lot # 9291059
14) Anti-CD031-eFluor 450; mouse monoclonal; clone WM59; eBioscience; 48-0319-42; lot # 1923228

16) Anti-Brachyury-APC; goat polyclonal; R&D; IC2085A; lot # AD4Q0115071
21) Anti mouse Ig FITC; goat polyclonal; BD Biosciences; 554001; lot # 7341616
22) Anti mouse lg-PE; goat polyclonal; BD Biosciences; 550589; lot # 7194652
23) Anti-CD90-PE; mouse monoclonal; clone 5E10; BD; 555596; lot # 28208
24) Anti-CD31-PE; mouse monoclonal; clone WM59; BD; 555446; lot # 42213
CDC42
CDC42 GTP

Validation

Antibodies for flow cytometry were validated in the Core Facility of the Paracelsus Medical University in Salzburg, including proper titration and controls.
Antibodies for histology were used according to the recommened manufacturer's protocol, with initial specificty test (specific human binding), positive and negative controls, isotype controls and second antibody only controls.

# Eukaryotic cell lines

Policy information about cell lines

Cell line source(s)

primary mesenchymal stem cells and fibroblasts were used from different donors.
iPSC were reprogrammed and characterized by the HSCI and registered at https://hpscreg.eu/cell-line/PMUi001 and PMUi002
Fibroblasts were isolated from Dock2 patients as described in Dobbs et al

Authentication

Permissions for human blood and marrow cell collection as well as genetic reprogramming were obtained from the Institutional Review Board Medical University of Graz (protocols EK 19–252, EK 21–060) and the Ethics Committee of the province of Salzburg (protocol 415-E/1776/4-2014). Samples were collected after written informed consent from healthy volunteers according to the Declaration of Helsinki.

Mycoplasma contamination

cell lines were not tested for Mycolpasma contamination

Commonly misidentified lines
(See ICLAC register)

Adult bone marrow- and neonatal umbilical cord blood-derived hiPSCs are described in a previous work (Peking et al. 2020).
Fibroblasts were isolated from patient material as descrubed in Dobbs et al

# Animals and other organisms

Policy information about studies involving animals; ARRIVE guidelines recommended for reporting animal research

Laboratory animals

No laboratory animals were used in this study.

Wild animals

No wild animals were used in this study.

Field-collected samples

No field-collected samples were used in this study.

Ethics oversight

No animal ethics was necessary for this sstudy.

Note that full information on the approval of the study protocol must also be provided in the manuscript.

# Flow Cytometry

## Plots

Confirm that:

- [x] The axis labels state the marker and fluorochrome used (e.g. CD4-FITC).
- [ ] The axis scales are clearly visible. Include numbers along axes only for bottom left plot of group (a 'group' is an analysis of identical markers).
- [ ] All plots are contour plots with outliers or pseudocolor plots.
- [x] A numerical value for number of cells or percentage (with statistics) is provided.

## Methodology

Sample preparation

After reaching 80% confluency, MSC, fibroblasts, hiPSCs, hiPSC-derived cell types types were detached with with 0.05% Trypsin/0.7 mM EDTA and centrifuge at 300g for 5 min at 4°C. The cells were resuspended to a concentratiom of 1x106 cells/mL in modified PBS and unspecific antibody binding was blocked with 10% v/v sheep serum for 20 min at 4°C. Then the corresponding concentration of antibody was added to the cells and incubated for 30 min at 4°C. After one washing step with PBS, the cells were analyzed using the flow cytometer.

Instrument

Five-laser BD LSR-FortessaTM (BD Bioscience) and  Gallios 10-color flow cytometer and the Kaluza G1.0 software (both Coulter)

Software | BD FACSDiva Software 8.0.1 Firmware version 1.4, Kaluza analysis software version 1.3.14026.13330 (Beckman Coulter)

Cell population abundance | *Describe the abundance of the relevant cell populations within post-sort fractions, providing details on the purity of the samples and how it was determined.*

Gating strategy | We applied forward and side scatter parameters (FSC, SSC) to exclude cell debris and doublets. Dead cells were excluded applying side scatter and dead stain.

☐ Tick this box to confirm that a figure exemplifying the gating strategy is provided in the Supplementary Information.
