# Supplementary material for: Extra-hematopoietic immunomodulatory role of the guanine-exchange factor DOCK2
Source: Commun Biol. 2022 Nov 15;5:1246. doi: 10.1038/s42003-022-04078-1 (PMC9666545; doi:10.1038/s42003-022-04078-1)
Supplement: Supplementary file 1 — Peer Review File [file 42003_2022_4078_MOESM1_ESM.pdf]

nature portfolio

Peer Review File

Open Access This file is licensed under a Creative Commons Attribution 4.0 International License, which permits use, sharing, adaptation, distribution and reproduction in any medium or format, as long as you give appropriate credit to the original author(s) and the source, provide a link to the Creative Commons license, and indicate if changes were made. In the cases where the authors are anonymous, such as is the case for the reports of anonymous peer reviewers, author attribution should be to 'Anonymous Referee' followed by a clear attribution to the source work. The images or other third party material in this file are included in the article's Creative Commons license, unless indicated otherwise in a credit line to the material. If material is not included in the article's Creative Commons license and your intended use is not permitted by statutory regulation or exceeds the permitted use, you will need to obtain permission directly from the copyright holder. To view a copy of this license, visit http://creativecommons.org/licenses/by/4.0/.

Reviewers' comments:

Reviewer #1 (Remarks to the Author):

In this study, the authors showed that guanine exchange factor DOCK2 is responsible for the development of immunomodulatory function in stromal cells (mesenchymal stem/progenitor cells (MSPC) and fibroblasts). The concept is examined by three models: 1) reprograming and redifferentiation of MSPCs, 2) fibroblasts obtained from SCID patients with DOCK2 mutation and 3) iPSC-MSPCs from DOCK2-knockout iPSCs. DOCK2 is known to modulate the cytoskeleton and this was also demonstrated in the study. DOCK2 is a relatively newly found gene related to immunity, therefore is an interesting target for MSC/MPSC immunomodulation. However, there are a number of concerns in the study before publication can be considered.

Comments:
1. The most critical question is whether DOCK2-mediated cytoskeletal regulation is involved in MSPC immunomodulation, as proposed in the manuscript but not answered. Immune assays should be performed on MSPCs modulated with cytoskeleton-related siRNAs or drugs to validate this hypothesis.
2. The authors have DOCK2-mutation patient fibroblasts, so why iPSCs from these patient-specific fibroblasts were not created and tested along with DOCK2-knockout iPSCs? Isn’t this the most important capacity of iPSCs ?
3. The study uses patient fibroblast in comparison to BM-, UC-, & iPSC-MPSCs. MPSCs/MSCs are distinct from fibroblasts in having tri-lineage differentiation capacity, so it is unclear why patient fibroblasts were used instead of MPSCs. An explanation within the manuscript is the least that should be done.
4. CD90 is one of the minimal criteria to characterize MSC but the iPSC-MSPCs showed no CD90 expression (Figure S1), indicating they could be immature MSPCs. This result may explain the restricted chondrogenic differentiation compared to parental BM-MSPCs. This should be discussed.
5. The authors highlighted iPSC-MSPCs that lacked immunosuppressive capability in previous studies which were summarized in Table S1, however, Wang et al. Stem Cells 2018 demonstrated with multiple sources of iPSC-MSCs that strong immunomodulation was present and similar to BMMSCs, and Bloor et al Nat Med 2020 has used iPSC-MSCs in a Phase 1 trial for GVHD. Please include these reference and revise text & table accordingly.
6. Cytoskeletal modulation is well known to lead to MSC differentiation; does DOCK2-mediated cytoskeletal modulation affect any MSC differentiation lineage?
7. Fig1B shows 16 markers, but the associated FigS1 does not show all markers.
8. Fig2C represent the gene lists of T-cell proliferation and G-protein signaling, but FigS7D and S7E do not show the associated results.
9. The abbreviation for human platelet lysate is inconsistent in the Materials/Methods scratch assay vs. FigS9c.
10. Please check Fig3 legend: it should be MSPC:PBMC ratio
11. Fig3: similar experiments should be presented similarly, i.e. for panels a & c, the T cell mitogenesis assay should be re-drawn to be presented similarly
12. Fig4a: no representative immunofluorescence figure is shown.
13. Fig4c: the legend mentions vicilin, but the photo is labeled CDC42.
14. Fig4 f, g, & h: Where are the Rx treated panels?
15. FigS5: there are negative controls in the osteogenic and adipogenic differentiation panels but not for chondrogenic differentiation.
16. Active CDC42-GTP pulldown Western blots Pulldown assay was mentioned in the manuscript but the data cannot be found.

Reviewer #2 (Remarks to the Author):

Stromal cells interact with immune cells during initiation and resolution of immune responses, though the precise underlying mechanisms remain to be resolved. The authors report on a novel function for the guanine exchange factor dedicator of cytokinesis-2 (DOCK2)3 in regulating the general immunosuppressive function in three human stromal cell models. To identify the immune function-related molecular signature in stromal cells, they reprogrammed mesenchymal stem/progenitor cells (MSPCs) into induced pluripotent stem cells (iPSCs) before differentiating these iPSCs in a back-loop into MSPCs. The iPSCs and immature iPS-MSPCs lacked immunosuppressive potential. Successive maturation facilitated emergence of immunomodulation, while maintaining clonogenicity, comparable to their parental MSPCs. Sequential transcriptomics and methylomics displayed time-dependent immune-related gene expression trajectories, including DOCK2, eventually resembling parental MSPCs. Severe combined immunodeficiency (SCID) patient-derived fibroblasts harboring bi-allelic DOCK2 mutations showed significantly reduced immunomodulatory capacity compared to non-mutated fibroblasts. Conclusively, CRISPR/Cas9-mediated DOCK2 knockout in iPS-MSPCs also resulted in significantly reduced immunomodulation, reduced CDC42 Rho family GTPase activation and blunted filopodia formation. The authors conclude that G protein signaling is a key element devising stromal cell immunomodulation.

This is an interesting paper using a novel approach to examine the emergence of transcriptional and functional aspects of immunomodulatory behavior of MSPCs. The data appear sound with robust statistical support. In the end, the main question remaining is how does the DOCK2 mutant cell loss of CDC42 activation and defective filopodia formation actually lead to diminished T cell proliferation? The authors should at least propose any published work that could suggest a plausible mechanism.

Reviewer #3 (Remarks to the Author):

In this paper, the authors show a novel function for the guanine exchange dedicator of cytokinesis 2 (DOCK2) in regulating the general immunosuppressive function in three human stromal cell models. They reprogrammed MSPCs into induced pluripotent stem cells (iPSCs) before differentiating these iPSCs in a back-loop into MSPCs. Using transcriptomics and methylomics, they highlight immune-related gene expression trajectories including DOCK2. Interestingly DOCK2-mutated fibroblast cell line from two SCID patients and DOCK2 knockout in iPS-MSCPs resulted in significant reduced immunomodulatory capacities. They conclude that G protein signaling is a key element devising stromal cell immunomodulation.

Overall the paper is very interesting and makes an important contribution to the field of immunomodulatory functions of MSPCs and their mode of action. This study is innovative because it relies on the use of "closed circles" of autologous oligoclonal MSPC-to-iPS-back-to-MSPC differentiation (derived from bone marrow and umbilical cord blood) to mimic stromal ontogeny. The paper appears of interest for the journal's readership. The manuscript needs only several minor revisions.

In the introduction, the authors say that "high expectations were not satisfied in clinical trials determining MSCPs immunomodulatory and trophic effects in part due to a limited understanding of their complex biology". However, the complexity of the mechanisms of action as well as their heterogeneity and plasticity depend on many other factors such as their tissue origin, culture conditions, inflammatory environment, the heterogeneity of administration protocols and patient characteristics contributing to the discrepancies between clinical studies. The authors should comment this point.

Canonical stromal marker upregulation as CD73 and CD105 associated to the reduction of Tra-1-81 was observed during the mesodermal differentiation of iPS. However, iPS-MSPCs were negative for CD90 and SSEA4 in contrast to the parental MSPCs. The authors should explain these discrepancies.

It has been reported that MSCs are not constitutively immunosuppressive but must be educated to

this behavior by specific environmental cues (inflammation, TLR challenging, hypoxia). Since DOCK2 functions can also dictate immunomodulatory stromal cell functions, do you think that these stimuli such as proinflammatory cytokines can also modulate the expression of DOCK2?

DOCK2 expression regulates MSPC immunomodulatory capacity acquisition during mesodermal stromal differentiation evidenced by the inhibition of T cell proliferation (mitogen and MLR). MSC are also capable of reprogramming macrophages into M2 phenotype characterized by the increased phagocytic ability, upregulated expression of anti-inflammatory cytokine IL-10, and suppressed expression of proinflammatory cytokines such as IL-12 and TNF-α. DOCK2 expression may also play a role in the ability of MSPCs to polarize macrophages. The authors should comment this point.

Salzburg, July 4, 2022

Dear reviewers:

Below you find the revised version of our manuscript COMMSBIO-22-0290-T on the "**Extra-hematopoietic immunomodulatory role of the guanine exchange factor DOCK2**". We have addressed all comments and criticisms. Further experiments were performed as suggested and results and comments have been included in the revised manuscript, as explained below in detail, and highlighted *in track mode* in the revised manuscript.

Reviewer #1 (Remarks to the Author):

In this study, the authors showed that guanine exchange factor DOCK2 is responsible for the development of immunomodulatory function in stromal cells (mesenchymal stem/progenitor cells (MSPC) and fibroblasts). The concept is examined by three models: 1) reprograming and redifferentiation of MSPCs, 2) fibroblasts obtained from SCID patients with DOCK2 mutation and 3) iPSC-MSPCs from DOCK2-knockout iPSCs. DOCK2 is known to modulate the cytoskeleton and this was also demonstrated in the study. DOCK2 is a relatively newly found gene related to immunity, therefore is an interesting target for MSC/MPSC immunomodulation. However, there are a number of concerns in the study before publication can be considered.

Comments:

1. The most critical question is whether DOCK2-mediated cytoskeletal regulation is involved in MSPC immunomodulation, as proposed in the manuscript but not answered. Immune assays should be performed on MSPCs modulated with cytoskeleton-related siRNAs or drugs to validate this hypothesis.

We tested three independent DOCK2 siRNAs and performed additional mitogenesis as well as MLR-based immunomodulation assays comparing iPS-MSPC and skin fibroblasts, with and without siRNA-mediated DOCK2 knockdown, respectively. Results confirmed that DOCK2 knockdown as confirmed by western blot (Fig.S10) results in a dose-dependent significant reduction of immunomodulatory function of both stromal cell types and are now included in the **new Fig. 3**.

2. The authors have DOCK2-mutation patient fibroblasts, so why iPSCs from these patient-specific fibroblasts were not created and tested along with DOCK2-knockout iPSCs? Isn't this the most important capacity of iPSCs ?

We agree that this would represent another interesting study. Initiating another IRB vote in two international hospitals to ask for permission of the parents of the two children which donated biopsy material for creating the patient fibroblast lines is out of the scope of this study. The patient fibroblast lines used in our study were originally obtained from two of five children in different European and US centers, suffering from severe combined immunodeficiency, in advance of transplantation (**https://www.nejm.org/doi/full/10.1056/NEJMoa1413462**). "Two patients died early in childhood; after allogeneic hematopoietic stem-cell transplantation, the other three had normalization of T-cell function and clinical improvement." Due to current European legal regulation, the generation of iPSCs would require new IRB votes to be submitted in the respective centers by the responsible transplant physician or pediatrician plus consent by the parents. As we are blinded to the identity of the cell donors, we are not aware of the fate of the children since transplantation. We thus also face the possibility to contact parents of a deceased patient. Furthermore, it is not clear whether we get access to the patient information required to contact the parents. Being a transplant physician, I am

aware that legal regulation in most countries prohibits disclosure of patient (and donor) identity outside transplant registries. The cells existing were obtained under an expired IRB vote before 2015. We are therefore not able to create new iPSC lines from existing patient material. We consider this point to be properly addressed.

3. The study uses patient fibroblast in comparison to BM-, UC-, & iPSC-MPSCs. MPSCs/MSCs are distinct from fibroblasts in having tri-lineage differentiation capacity, so it is unclear why patient fibroblasts were used instead of MPSCs. An explanation within the manuscript is the least that should be done.

Following this reviewer's request, we now state in the revised manuscript that "fibroblast lines were selected as representing skin stromal cells from DOCK2 SCID patients". Immunomodulation is a common function of most if not all stromal cells, including fibroblasts (e.g., https://www.ncbi.nlm.nih.gov/pmc/articles/PMC7550172). A more general discussion about stromal cell terminology is out of the focus of this manuscript.

We may add at this point that there is meanwhile common agreement in the scientific community that the in vitro differentiation assays "… are highly prone to artifact or misinterpretation. For the osteogenesis assay, alizarin red S cannot distinguish between dystrophic calcification induced by dead and dying cells versus matrix mineralization. Furthermore, if the cells make the enzyme alkaline phosphatase, it cleaves β-glycerophosphate, a component of osteogenic differentiation medium" (cited from https://pubmed.ncbi.nlm.nih.gov/28491279/). It is therefore not surprising that many papers found also skin-derived fibroblasts to respond in these so-called tri-lineage differentiation assays (e.g., https://pubmed.ncbi.nlm.nih.gov/22384246/). An example for an artifact in chondrogenic differentiation analysis can be found in the new Table S1 reference 29 requested to be included by this reviewer, showing cell aggregates without any evidence of chondrogenesis in Fig.1D.

4. CD90 is one of the minimal criteria to characterize MSC but the iPSC-MSPCs showed no CD90 expression (Figure S1), indicating they could be immature MSPCs. This result may explain the restricted chondrogenic differentiation compared to parental BM-MSPCs. This should be discussed.

Regarding CD90 expression we are aware that iPS-MSPC at passage eight as shown in Fig.1 lack CD90 that was highly expressed by their predecessor iPSC (Fig.S1). On p.5 of the revised manuscript we therefore highlight "the lack of complete surface marker identity, e.g., diminished CD90. Further phenotypic maturation was achieved beyond p8 (see also Ref.30) (Fig.9c)…" In Table S1 we also highlight that lack of CD90 or delayed expression is a common phenomenon (e.g. found in Table S1 references 7, 19, 21, 25)

Regarding the so-called "MSC-criteria" we may point again to the common agreement in the stem cell community to abandon the incorrect term "MSC" (see also https://media.nature.com/original/magazine-assets/d41586-018-06756-9/d41586-018-06756-9.pdf). We may add here that CD90 is an established marker of fibroblasts in various tissues (e.g., https://www.ncbi.nlm.nih.gov/pmc/articles/PMC6204438/). In this context we also like to draw the attention of this reviewer to Fig.S9a confirming

homogenous expression of the fibroblast marker CD90 on all patient-derived as well as all control fibroblasts.

5. The authors highlighted iPSC-MSPCs that lacked immunosuppressive capability in previous studies which were summarized in Table S1, however, Wang et al. Stem Cells 2018 demonstrated with multiple sources of iPSC-MSCs that strong immunomodulation was present and similar to BMMSCs, and Bloor et al Nat Med 2020 has used iPSC-MSCs in a Phase 1 trial for GVHD. Please include these reference and revise text & table accordingly.

Both references were included in Table S1 and the text was revised accordingly.

6. Cytoskeletal modulation is well known to lead to MSC differentiation; does DOCK2-mediated cytoskeletal modulation affect any MSC differentiation lineage?

We agree with the reviewer that actin and actin disruption modulate the differentiation of stromal cells presumably including their clonogenic progenitors, i.e. MSPCs. Indeed, in vitro disruption of F-actin with cytochalasin has been reported to reduce osteogenesis in a p38-dependent manner (Sonowal et al. J Biomed Sci. 2013; 20:71, https://pubmed.ncbi.nlm.nih.gov/24070328/). However, others report that cytochalasin D promotes osteogenesis, by stimulating recruitment of nuclear ARP2/3 to G actin (Sen et al. Stem Cells. 2017; 35: 1624, https://pubmed.ncbi.nlm.nih.gov/28371128/). Our own in vivo work suggests that, reduced F-actin may drive the loss of mature adipocytes over time (Landspersky et al. Blood. 2022; 139: 690, https://pubmed.ncbi.nlm.nih.gov/34657154/). This information was now included in the discussion in the revised manuscript. Further investigation of this aspect was out the focus of the current study.

7. Fig1B shows 16 markers, but the associated FigS1 does not show all markers.

We now display all 16 markers shown in Fig.1B in a revised Fig.S1 as requested.

8. Fig2C represent the gene lists of T-cell proliferation and G-protein signaling, but FigS7D and S7E do not show the associated results.

Figure 2c shows RNA-seq results whereas Fig.S7d,e show methylation data. Methylation analysis revealed that not all of the differentially expressed genes are differentially methylated. Therefore, the list of genes displayed in both figures differs. A detailed description of these results can be found on page 6+7.

9. The abbreviation for human platelet lysate is inconsistent in the Materials/Methods scratch assay vs. FigS9c.

Thank you. Corrected accordingly to hPL in the scratch assay methods on p.16 and in Fig.S9c.

10. Please check Fig3 legend: it should be MSPC:PBMC ratio

Corrected accordingly, including also the correct X-axis title to the new data (see above).

11. Fig3: similar experiments should be presented similarly, i.e. for panels a & c, the T cell mitogenesis assay should be re-drawn to be presented similarly

Corrected accordingly.

12. Fig4a: no representative immunofluorescence figure is shown.

Representative immunofluorescence Fig.S12 was included as requested and we also refer to these results in the legend to Fig.4.

13. Fig4c: the legend mentions vicilin, but the photo is labeled CDC42.

Thank you – corrected accordingly.

14. Fig4 f, g, & h: Where are the Rx treated panels?

We added a revised Fig. 4 with f, g, and h now showing the respective treatments as heading to the figure.

15. FigS5: there are negative controls in the osteogenic and adipogenic differentiation panels but not for chondrogenic differentiation.

Negative control for chondrogenesis added as requested.

16. Active CDC42-GTP pulldown Western blots Pulldown assay was mentioned in the manuscript but the data cannot be found.

CDC42 pulldown data were now added as requested in a new Figure S11. We now show results from n = 6 experiments resulting in 1.95-fold reduction of active CDC42-GTP in the patient fibroblasts compared to the control fibroblasts, and mean 3.87-fold CDC42-GTP reduction in the DOCK2-/- iPSCs (0.71) compared to the wild-type iPSCs (2.77).

Reviewer #2 (Remarks to the Author):

Stromal cells interact with immune cells during initiation and resolution of immune responses, though the precise underlying mechanisms remain to be resolved. The authors report on a novel function for the guanine exchange factor dedicator of cytokinesis-2 (DOCK2)3 in regulating the general immunosuppressive function in three human stromal cell models. To identify the immune function-related molecular signature in stromal cells, they reprogrammed mesenchymal stem/progenitor cells (MSPCs) into induced pluripotent stem cells (iPSCs) before differentiating these iPSCs in a back-loop into MSPCs. The iPSCs and immature iPS-MSPCs lacked immunosuppressive potential. Successive maturation facilitated emergence of immunomodulation, while maintaining clonogenicity, comparable to their parental MSPCs. Sequential transcriptomics and methylomics displayed time-dependent immune-related gene expression trajectories, including DOCK2, eventually resembling parental MSPCs. Severe combined
immunodeficiency (SCID) patient-derived fibroblasts harboring bi-allelic DOCK2 mutations showed significantly reduced immunomodulatory capacity compared to non-mutated fibroblasts. Conclusively, CRISPR/Cas9-mediated DOCK2 knockout in iPS-MSPCs also resulted in significantly reduced immunomodulation, reduced CDC42 Rho family GTPase activation and blunted filopodia formation. The authors conclude that G protein signaling is a key element devising stromal cell immunomodulation.
This is an interesting paper using a novel approach to examine the emergence of transcriptional and functional aspects of immunomodulatory behavior of MSPCs. The data appear sound with robust statistical support. In the

end, the main question remaining is how does the DOCK2 mutant cell loss of CDC42 activation and defective filopodia formation actually lead to diminished T cell proliferation? The authors should at least propose any published work that could suggest a plausible mechanism.

Thank you for highlighting this point. Following this reviewer's recommendation, we included the following paragraph prominently at the end of the discussion:

DOCK2 as a GEF functions as an activator of CDC42. We found significantly reduced CDC42-GTP in pulldown assays in *DOCK2*-/- compared to unmutated stromal cells. Activated CDC42-GTP drives condensation of the Arp2/3 complex required for F-actin polymerization, the actin-myosin network and filopodia formation. While the network is an important transporting vehicle for both endo- and exocytosis, the filopodia are important for synapse formation with interacting cells, such as T cells. Our results are consistent with the view that DOCK2 deficiency reduces CDC42 activation as well as the formation of F-actin filaments and filopodia. Considering the reduced immunomodulation by DOCK2-deficient stromal cells, we speculate that the stromal cells show either reduced endo- and exocytosis or reduced synapse formation with interacting cells, or both.

Reviewer #3 (Remarks to the Author):

In this paper, the authors show a novel function for the guanine exchange dedicator of cytokinesis 2 (DOCK2) in regulating the general immunosuppressive function in three human stromal cell models. They reprogrammed MSPCs into induced pluripotent stem cells (iPSCs) before differentiating these iPSCs in a back-loop into MSPCs. Using transcriptomics and methylomics, they highlight immune-related gene expression trajectories including DOCK2. Interestingly DOCK2-mutated fibroblast cell line from two SCID patients and DOCK2 knockout in iPS-MSCPs resulted in significant reduced immunomodulatory capacities. They conclude that G protein signaling is a key element devising stromal cell immunomodulation.

Overall the paper is very interesting and makes an important contribution to the field of immunomodulatory functions of MSPCs and their mode of action. This study is innovative because it relies on the use of "closed circles" of autologous oligoclonal MSPC-to-iPS-back-to-MSPC differentiation (derived from bone marrow and umbilical cord blood) to mimic stromal ontogeny. The paper appears of interest for the journal's readership. The manuscript needs only several minor revisions.

In the introduction, the authors say that "high expectations were not satisfied in clinical trials determining MSCPs immunomodulatory and trophic effects in part due to a limited understanding of their complex biology". However, the complexity of the mechanisms of action as well as their heterogeneity and plasticity depend on many other factors such as their tissue origin, culture conditions, inflammatory environment, the heterogeneity of administration protocols and patient characteristics contributing to the discrepancies between clinical studies. The authors should comment this point.

We agree with this criticism and realize that "limited understanding of their complex biology by far does not cover all reasons of the limited success of the thousands of stromal cell ('MSC') trials. In order not to distract the readers' attention too much from the topic of DOCK2 and immunomodulation, we consequently deleted the explanation.

Canonical stromal marker upregulation as CD73 and CD105 associated to the reduction of Tra-1-81 was observed during the mesodermal differentiation of iPS. However, iPS-MSPCs were negative for CD90 and SSEA4 in contrast to the parental MSPCs. The authors should explain these discrepancies.

Thank you for making this point. As described in response to reviewer 1's question #4, we were aware that iPS-MSPC at passage eight as shown in Fig.1 lack CD90 that was highly

expressed by their predecessor iPSC (Fig.S1). On p.5 of the revised manuscript, we now highlight “the lack of complete surface marker identity, e.g., diminished CD90 and SSEA-4. Further phenotypic maturation was achieved beyond p8 (see also Ref.30) (Fig.9c)...” In Table S1 we also highlight that lack of CD90 or delayed expression is a common phenomenon (e.g. found in Table S1 references 7, 19, 21, 25). Concerning SSEA-4 we may add that we observed high variability in different stomal cell populations and preparations over the last decades. SSEA-4 was not studied in more detail in the further course of the study.

It has been reported that MSCs are not constitutively immunosuppressive but must be educated to this behavior by specific environmental cues (inflammation, TLR challenging, hypoxia). Since DOCK2 functions can also dictate immunomodulatory stromal cell functions, do you think that these stimuli such as proinflammatory cytokines can also modulate the expression of DOCK2?

Thank you for making this point. We worked hard over several years and were unfortunately not able so far to establish single cell-based DOCK2 quantification technology. The original antibody used in the Dobbs ... Notarangelo_2015_NEJM article (Ref.#3) is no longer available. We tested multiple different antibodies and identified one clone (Thermo Fisher, Cat.MA5-26547) that could be titrated to obtain acceptable quality semi-quantitative Western blot bulk analysis results (Fig.S10), but does not allow for more sensitive quantification of DOCK2 expression. We therefore opted to perform additional immunomodulation experiments testing the response of *DOCK2*Δ/Δ patient-derived fibroblast lines as compared to control fibroblasts, to TNF-α plus IFN-γ pro-inflammatory signaling. The fact that DOCK2-mutated SCID patient-derived fibroblasts also respond to IFN/TNF signals might rather argue in favor of additional signaling pathways rather than elevated DOCK2 expression. Results are now included in the new Fig.S14 and showed significant dose-dependent increase of immunomodulatory capacity. These data are in line with previous results showing reduced virus-induced cytotoxicity in the presence of IFN-α in the original NEJM article. This observation is now discussed in the revised manuscript.

DOCK2 expression regulates MSPC immunomodulatory capacity acquisition during mesodermal stromal differentiation evidenced by the inhibition of T cell proliferation (mitogen and MLR). MSC are also capable of reprogramming macrophages into M2 phenotype characterized by the increased phagocytic ability, upregulated expression of anti-inflammatory cytokine IL-10, and suppressed expression of proinflammatory cytokines such as IL-12 and TNF-α. DOCK2 expression may also play a role in the ability of MSPCs to polarize macrophages. The authors should comment this point.

Thank you. A comment was included in the discussion accordingly in the before-last paragraph.

REVIEWERS' COMMENTS:

Reviewer #1 (Remarks to the Author):

Major comments:
1. Representative data in the new Figure 3 should be given since T-cell inhibition was decreased with increased fibroblast or MSPC numbers and enhancement of T-cell proliferation was unexpectedly shown with fibroblast or MSPC treatment in several results. Moreover, please describe the data in more detail, e.g., the statistical difference between compared groups in Figure 3d.
2. The sentence on p.4 “A setback in previous studies was that iPSC-derived MSPCs mostly lacked immunosuppressive capacity (summarized in Table S1)” is misleading and needs to be revised to reflect the fact that iPSC-MSCs are currently used in clinical trials to suppress inflammatory/immune conditions, especially since such references have now been listed/cited in Table S1.
3. The explanation on the logistical and ethical difficulties in obtaining patient-derived fibroblasts to create disease-specific iPSCs is appreciated, and should be very briefly mentioned in the manuscript to provide readers with insight on the challenges of obtaining patient samples.
Minor comment: in Figure 3, the ratios are labeled 1:1, 1:3 and 1:9 on the figure, but in the legend it is written as 1:1, 1:3, 1:10.

Reviewer #2 (Remarks to the Author):

The authors have addressed all my concerns.

Reviewer #3 (Remarks to the Author):

The authors have satisfactorily answered my questions. This article is now publishable in this form in "Communications Biology".

Salzburg, August 19, 2022

Dear reviewers:

Thank you for your efforts helping to complete a finally revised version of our manuscript COMMSBIO-22-0290-T on the "**Extra-hematopoietic immunomodulatory role of the guanine exchange factor DOCK2**". We have addressed all comments of reviewer #1, as explained below in detail, and highlighted *in red* in the revised manuscript.

Reviewer #1 (Remarks to the Author):

1. Representative data in the new Figure 3 should be given since T-cell inhibition was decreased with increased fibroblast or MSPC numbers and enhancement of T-cell proliferation was unexpectedly shown with fibroblast or MSPC treatment in several results. Moreover, please describe the data in more detail, e.g., the statistical difference between compared groups in Figure 3d. Minor comment: in Figure 3, the ratios are labeled 1:1, 1:3 and 1:9 on the figure, but in the legend it is written as 1:1, 1:3, 1:10.

Thank you for the identification of this unpleasant error – we mistyped the ratios FB:PBMC & MSPC:PBMC and results were as expected with declining inhibition of T cell proliferation at lower input stromal cell number, now corrected accordingly in the **new Fig. 3**. Also the imprecise 1:10 label in the legend was corrected to 1:9 !

2. The sentence on p.4 "A setback in previous studies was that iPSC-derived MSPCs mostly lacked immunosuppressive capacity (summarized in Table S1)" is misleading and needs to be revised to reflect the fact that iPSC-MSCs are currently used in clinical trials to suppress inflammatory/immune conditions, especially since such references have now been listed/cited in Table S1.

Changed as requested. The new sentence reads: "The stromal cell-inherent immunosuppressive capacity was analyzed in some but not all previous studies on iPSC-derived MSPCs (summarized in **Table S1**)."

3. The explanation on the logistical and ethical difficulties in obtaining patient-derived fibroblasts to create disease-specific iPSCs is appreciated, and should be very briefly mentioned in the manuscript to provide readers with insight on the challenges of obtaining patient samples.

Comments included in the revised manuscript on page 14.

Thank you for your thorough review.

Yours sincerely

D.Strunk
